# Structural, Mechanical, and Tribological Properties of Oriented Ultra-High Molecular Weight Polyethylene/Graphene Nanoplates/Polyaniline Films

**DOI:** 10.3390/polym15030758

**Published:** 2023-02-02

**Authors:** Tarek Dayyoub, Aleksey Maksimkin, Leonid K. Olifirov, Dilus Chukov, Evgeniy Kolesnikov, Sergey D. Kaloshkin, Dmitry V. Telyshev

**Affiliations:** 1 Department of Physical Chemistry, National University of Science and Technology “MISIS”, 119049 Moscow, Russia; 2 Institute for Bionic Technologies and Engineering, I.M. Sechenov First Moscow State Medical University (Sechenov University), Bolshaya Pirogovskaya Street 2-4, 119991 Moscow, Russia; 3 Institute of Biomedical Systems, National Research University of Electronic Technology, Zelenograd, 124498 Moscow, Russia

**Keywords:** UHMWPE, polyethylene wax, graphene nanoplates, polyaniline, lamellar structure, films, cavitation, orientation hardening, mechanical properties, work of fracture, coefficient of friction, wear

## Abstract

Preparing high-strength polymeric materials using an orientation drawing process is considered one of the most urgent topics in the modern world. Graphene nanoplates/polyaniline (GNP/PANI) were added to the commercial grade UHMWPE (GUR 4120) matrix as a filler with antifriction properties. The effect of GNP/PANI addition on the structure, the orientation process, the void formation (cavitation), the mechanical, and tribological properties was studied using differential scanning calorimetry (DSC), dynamical mechanical analysis (DMA), and scanning electron microscopy (SEM). The paper’s findings indicated an increase in the cavitation effect of 120–320% after the addition of GNP/PANI to the UHMWPE polymer matrix. This increase, during the process of the oriented films’ thermal orientation hardening, led, in turn, to a decrease in the tensile strength during the process of the oriented films’ thermal orientation hardening. Furthermore, the decrease in the coefficient of friction in the best samples of oriented UHMWPE films was two times greater, and the increase in wear resistance was more than an order of magnitude. This process was part of the orientation hardening process for the UHMWPE films containing PE-wax as an intermolecular lubricant, as well as the presence of GNP/PANI in the material, which have a high resistance to abrasive wear.

## 1. Introduction

Using the orientation drawing process to obtain oriented films and fibers based on commercially available and cheap polymer grades is considered an interesting scientific direction. However, understanding the mechanisms of the orientation hardening process will allow one to achieve highly oriented polymers with improved mechanical and tribological properties, as well as minimizing the effect of cavitation, which in turn will make it possible to achieve a significant increase in the operational characteristics of polymeric products. Polymers play an important role not only in industrial applications, but also in medical and aerospace applications, particularly when unique tribological properties are required. This is related to their unique properties such as their low cost, easy processing, high corrosion resistance, low friction, and damping of noise and vibrations [1]. Ultra-high molecular weight polyethylene (UHMWPE) is one of the most important polymers and is used in a lot of applications. UHMWPE in its isotropic state has high wear resistance, a low coefficient of friction, self-lubricating properties, high chemical inertness, good strength, and biocompatible properties [2,3,4,5,6]. However, UHMWPE has some critical limitations that do not allow it to be fully used as an engineering material. Bulk UHMWPE with an isotropic structure state has low values of both modulus of elasticity and yield strength, and it exhibits high creep under constant and high loads. In terms of its tribological properties, UHMWPE is superior to nylon and polyformaldehyde, which are widely used in applications requiring a low coefficient of friction [7,8,9,10]. UHMWPE has a friction coefficient close to that of polytetrafluoroethylene, but UHMWPE surpasses it in wear resistance [11]. 

On the other hand, using the drawing and orientation hardening process to enhance the mechanical properties of the UHMWPE and increase its crystallinity can be the most effective way to improve its tribological properties [12]. Moreover, adding some second-phase particles, which have good tribological properties, into a UHMWPE matrix to form UHMWPE composites can also be an interesting improvement method [13].

UHMWPE has a high molecular weight of more than 10^6^ g/mol, which makes its melt flow index extremely low. This is related to the presence of the entanglements in the UHMWPE amorphous phase in a large number; and they will block the UHMWPE chains’ movement during the drawing process. This obstacle will cause a premature rupture in the UHMWPE macromolecules during the orientation process and prevent obtaining a high degree of effective orientation [14,15]. Therefore, adding low molecular weight polyethylene, such as PE-wax, to the UHMWPE matrix will bring down the melt viscosity and facilitate the drawing process of the UHMWPE films [16,17]. UHMWPE with a highly oriented structure has excellent mechanical and tribological properties, making it one of the best materials for industrial and medical applications, where high strength and low friction materials are needed [18,19]. The oriented films based on the commercial UHMWPE brands are lightweight, with high tensile strength values up to 1.5 GPa, high Young’s modulus values up to 40 GPa, and a highly crystallized structure up to 98% [17,20]. Edoumy et al. reported a decrease in wear of the oriented UHMWPE in comparison with the non-oriented UHMWPE when the samples were tested in directions parallel and perpendicular to the UHMWPE chain direction. In dry friction, the UHMWPE chains are oriented on the surface of the sample, which increases the wear resistance of the material. The wear surfaces of the oriented and non-oriented UHMWPE have a wavy morphology. The wavelength is proportional to the thickness of the lamella, which, in turn, decreases with the orientation of the UHMWPE. The friction film thickness is thinner in the case of the oriented UHMWPE [12]. In reference [21], they reported that the UHMWPE wear value strongly depends on the type of sliding motions. They found that during unidirectional sliding motion, UHMWPE macromolecules are oriented in the direction of motion. As a result, the UHMWPE surface becomes more durable and wear-resistant. In contrast, during multi-directional sliding motion, the UHMWPE chains had not been arranged, and the orientation processes of macromolecules on the UHMWPE surface were weakly expressed, which explains the increased wear values of the UHMWPE. In reference [22], they investigated the influence of the UHMWPE molecular orientation on the friction and wear behavior when sliding along the rough surfaces of the counter-faces. They found that, under abrasive conditions as well as in conditions of multidirectional movement, oriented UHMWPE does not have significant advantages in comparison with isotropic UHMWPE. Additionally, Chang et al. reported that the difference in wear resistance between the oriented and the non-oriented UHMWPE was insignificant [23]. In reference [24], a wear model was developed that allowed for the study of the changes in the UHMWPE molecular structure under the multidirectional stresses on the articular surface. The authors reported that in the process of the unidirectional motion during friction, the UHMWPE molecular structure will be reorganized under the stress fields. The molecules are stretched along the sliding direction, which leads to hardening of the surface of the polymer. In the case of multidirectional motions, the UHMWPE surface will be softened, and its wear resistance will be decreased.

However, the addition of low molecular weight material, such as PE-wax as an intermolecular lubricant, can improve the mechanical properties of the oriented UHMWPE films obtained by applying the orientation hardening process, but with its effect as a lubricant, it can lead to a reduction in the wear resistance of the films [25]. Therefore, the addition of friction-reducing material to the oriented UHMWPE, such as carbon fillers, can decrease the friction force and improve the wear resistance of these films [26,27,28,29,30]. Moreover, these carbon fillers can facilitate the drawing and orientation processes and increase the maximum obtained draw ratio [26]. In reference [27], they reported that the increase in the graphene nanoplatelet (GNP) content in the UHMWPE matrix led to an improvement in the mechanical properties of the UHMWPE/GNP composites, and this led to an improvement in the coefficient of friction and the wear resistance by four times due to the lubrication and toughening effects of the GNP and the easy shear of GNP. They reported that, by adding 1 wt. % of GNP, the tensile strength value was increased from 45 MPa up to 68 MPa in comparison with virgin UHMWPE. This improvement in mechanical properties led to a decrease in wear loss due to lubrication caused by the inter-layer shearing between the GNP layers. In reference [28], they confirmed that the addition of graphene oxide nanosheets (GO) by 1.0 wt. % led to significantly improving the hardness and wear resistance of the UHMWPE/GO composites, whereas the coefficient of friction was lightly increased. They also reported that the tribological behavior of these composites changed from fatigue wear to abrasive wear after the GO addition. This was related to the formation of a tribolayer on the contact wear surface. In reference [29], they prepared UHMWPE/GO nanocomposites using two methods. The first one was by the exfoliation of the graphene oxide (GO) in organic solvent and reducing it to graphene before the polymer addition, while the second one was by GO reduction after the polymer addition using the in situ reduction method. They confirmed that UHMWPE/GO films prepared by the pre-reduction method had higher crystallinity, better mechanical properties, and improved creep resistance in comparison with the in situ reduction method and virgin UHMWPE films.

**Figure 1:**
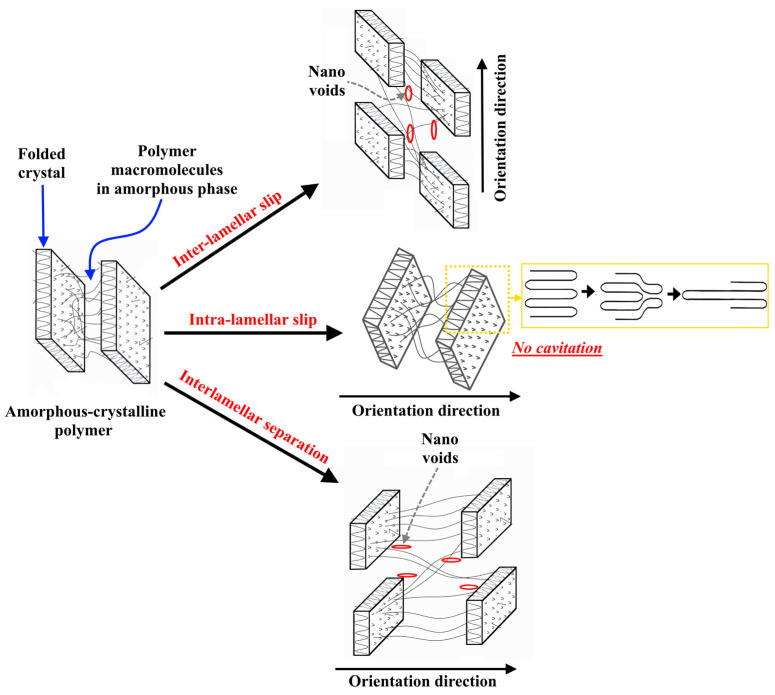
Deformation mechanisms of amorphous-crystalline polymer.

For many years, the cavitation effect was only associated with discontinuity processes in low molecular weight liquids [31,32]. The cavitation that occurs in low-molecular weight liquids is affected by the following factors: (a) the magnitude of the resulting tensile stresses and their relationship with the tensile strength of the low-molecular weight liquid itself; (b) the physical and thermodynamic parameters of the liquid; and (c) the presence of cavitation nuclei in the form of vapor bubbles, gases, or solid impurities. In the case of amorphous–crystalline polymers, the phenomenon of the cavitation effect is observed during crystallization from the melt [33,34,35] and during tensile deformation [36,37]. The cavitation effect in amorphous and crystalline polymers is driven by the negative pressure generated in the material, as in low molecular weight liquids. Negative pressure can be generated during the crystallization in areas densely surrounded by growing spherulites, and the cavitation bubbles are mainly observed in these regions. When polymers are stretched, cavities can form in the inter-spherulite regions and inside the spherulites themselves, which makes this phenomenon more intense, and it is initiated at an early stage of deformation (after reaching the yield point). If the deformation of the amorphous–crystalline polymer is accompanied by cavitation, then it is localized in its amorphous phase. In some cases, the properties of the amorphous phase of the polymer at a temperature above its glass transition temperature can be similar to those of low-molecular-weight liquids. The essential difference is the presence of passing macromolecules between crystallites (tie molecules and cilia) and the physical links (entanglements) between macromolecules in the polymer amorphous phase, which significantly limit the mobility of macromolecules. It should be noted that the deformation of amorphous–crystalline polymers occurs in accordance with three main mechanisms: intra-lamellar slip, interlamellar slip, and separation, as well as rotation of lamella stacks [38,39]. The deformation caused by the mechanism of intra-lamellar slip occurs only in the crystalline phase of the polymer due to the sliding of folded macromolecules located in crystals along the planes, which leads to the orientation of macromolecules and the formation of a fibrillar structure, which consists of stretching macromolecules in the direction of stretching (Figure 1). The main feature of this mechanism is the formation of an orientational crystal structure without the effect of cavitation, i.e., without the formation of voids in the amorphous phase of the oriented polymer. The deformation of the amorphous–crystalline polymer by the mechanisms of inter-lamellar slip and separation leads to the formation of a cavitation effect. These mechanisms lead to a change in the distances between adjacent lamellae when the direction of applied tensile stress is perpendicular to the lamella surface (Figure 1). The intensity of the flow of polymer deformation by the mechanism of inter-lamellar slip is influenced by the material’s structure, particularly the number and distribution of tie macromolecules between crystallites, as well as the size of the crystalline phase. As a result of the lamellae separation in the volume of the elongated polymer, an intensive process of void formation will occur [40]. It should be noted that, for materials that are characterized by a relatively high modulus of elasticity and the ability to undergo significant plastic deformation (UHMWPE and high-density polyethylene), the process of separation of lamellae is accompanied by local changes in the density of the sample, the result of which is the formation of material inhomogeneity (voids). Visually, this effect is expressed in a strong whitening of the sample upon reaching high degrees of drawing. In references [41,42], the authors demonstrated a strong dependence of the manifestation of the cavitation effect on the strain rate for isotactic polypropylene. As the strain rate increases, so do the stresses at which the polymer reaches its yield strength. This means that the crystalline phase is subjected to high stresses, which increase the cavitation effect. It was noted in reference [43] that the increase in the molecular weight of a polymer promotes an increase in the density of the physical entanglements of macromolecules. Physical links (entanglements) in the amorphous phase are considered a reinforcing element that increases the strength of the amorphous phase and, therefore, reduces the effect of cavitation. In addition, the deformation of polymers with large, strong, and defect-free crystals, such as polypropylene (PP), high-density polyethylene (HDPE), and UHMWPE, is accompanied by intense cavitation processes. If the polymer has a finely crystalline and defective structure, such as low-density polyethylene (LDPE), deformation occurs with significantly less cavitation. This is because the plastic deformation of small and defective crystals begins at lower stresses, which do not exceed the strength of the amorphous phase [44]. This means that cavitation is more intense in polymers with high crystalline strength because most of the applied stresses in the orientation process are transferred to the amorphous phase [44]. The type of applied stress (tension, compression, or shear) on the polymer plays a key role in the appearance of cavitation [45]. Under uniaxial tension, the deformation of the crystalline phase is predominantly carried out by the mechanism of interlamellar slip, which is accompanied by rupture and slip of macromolecules in the amorphous phase. Under compressive and shear stresses, the deformation of the crystalline phase mainly occurs according to the mechanism of intra-lamellar slip, and the orientation of macromolecules occurs without the formation of any cavities.

It should be noted that, in our previous article [20], the used UHMWPE had a molecular weight of 10^6^ g/mole and was considered a special grade compared to the available commercial grade that was used in this article (GUR 4120). This lower molecular weight makes the processing of the polymer and orientation hardening easier in comparison with other UHMWPE grades, such as the UHMWPE grade used in this article, which has an average molecular weight of 5 × 10^6^ g/mol. This difficulty in the orientation process using a UHMWPE grade with a high molecular weight is related to the presence of a large number of entanglements in the UHMWPE amorphous phase, which blocks the UHMWPE chains’ movement during the orientation process, as was mentioned above. This will cause a high level of stress on the polymer macromolecules in the amorphous phase, leading to their premature rupture during the orientation process. On the other hand, the role of the carbon fillers in the highly oriented polymeric films on cavitation is weakly studied in the published articles. Currently, and based on the above-mentioned literature review, the majority of published articles investigated the influence of carbon fillers on the mechanical and tribological properties of polymers with an isotropic structure, with a few investigating this influence on polymers with an oriented structure. However, all published articles are deficient in discussing the mechanical and tribological properties of oriented polymer/carbon filler films in terms of structure and cavitation. Therefore, in this article, we tried to focus on these points.

This work is aimed at studying the influence of GNP/PANI addition on the oriented macromolecular structure, cavitation, and mechanical properties of the UHMWPE composites with PE-wax and the influence of GNP/PANI addition on the tribological properties of the UHMWPE films.

## 2. Materials and Methods

### 2.1. Materials

UHMWPE GUR 4120 with a molecular weight of 5 × 10^6^ g/mol was purchased from “Ticona GmbH” (Frankfurt, Germany); and polyethylene wax PLWN-3W with a molecular weight of 4000 g/mol was purchased from “INHIMTEK LLC” (Novokuibyshevsk, Russia). Graphene nanoplates (GNP) were obtained by oxidative intercalation of expanded graphite with subsequent ultrasonic treatment and purchased from Nanotechcenter Ltd. (Tambov, Russia). GNP was functionalized by the deposition of polyaniline (PANI) on the GNP surface because of the oxidative polymerization of aniline. All procedures and conditions were broadly explained and described in the reference [20]. Figures S1–S4 present the SEM, TEM, and Raman spectra for both unmodified GNP and modified GNP by PANI. p-xylene was used as a plasticizer for the UHMWPE composites at a ratio of 2.5 mL of a solvent per 1 g of the polymer blend.

### 2.2. Fabrication of the Films

First, it should be noted that applying the drawing and orientation processes to the UHMWPE GUR 4120/GNP/PANI composites is very difficult. Technically, no normal UHMWPE GUR 4120/GNP/PANI films were obtained. Therefore, the addition of the PE-wax as an intermolecular lubricant to the UHMWPE GUR 4120 matrix to enhance its processability is very important for obtaining normally oriented films. Depending on the results presented in our previous work [17], PE-wax was added at a content of 1 wt. % for all polymer composites. 

Using a high-energy planetary ball mill (APF-3), mixing UHMWPE, PE-wax, and GNP/PANI powders was conducted in steel drums with a volume of 900 mL. Steel balls (Russian grade SHKH15) with a diameter of 7 to 9.5 mm were used as grinding media, and the average rotation speed of the carrier was 450 rpm. The temperature of the blends was kept at 60 °C during the milling process by using current water to cool the steel drums. The total time for mixing was 90 min. The UHMWPE/PE-wax/GNP/PANI powders with various GNP/PANI contents (0.01, 0.1, 0.5, 1.0, 2.0, 4.0 wt. %) were prepared.

Using a small amount of the solvent as a plasticizer, gel-spinning technology was used to obtain oriented UHMWPE films [46]. First, the UHMWPE/PE-wax/GNP/PANI blends were stirred with p-xylene at 140 ± 2 °C for 20 min. The UHMWPE/p-xylene gel was then extruded at 150 ± 3 °C using a ram extruder from UE-MSL (Extrusion Machinery Sales Ltd., Liversedge, UK) after being stored at this temperature in the extruder for 15 min. The used die size was 10 × 2 mm, and the extrusion rate was equal to 500 mm/min. Then, to obtain the xerogel (solvent-free UHMWPE gel), the extruded gel was dried for 48 h at room temperature.

**Figure 2:**
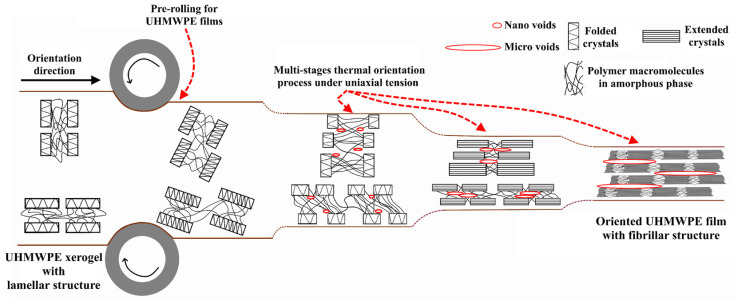
Proposed diagram that illustrates the preparation stages of the oriented UHMWPE films using the thermal orientation hardening process.

To reduce cavitation during the thermal orientation process and avoid the formation of voids in the polymer structure at the beginning of the orientation process, UHMWPE xerogels were preliminary rolled at a temperature of 110 °C until a deformation of 100% was achieved, as shown in Figure 2. During rolling, the deformation of the crystalline phase of UHMWPE proceeds under shear stresses, which activates the intra-lamellar slip mechanism, which is not accompanied by the cavitation effect. This means that, during the rolling process, shear slip of crystallographic planes along the [100] plane in the direction of the main chain [001] occurs, which means that this slip is considered a result of thin slip of linear defects (screw dislocation) along the slip plane. As a result of the intra-lamellar slid mechanism, the thickness of the folded lamellae increases.

In the drawing process and by applying tensile stresses at a drawing temperature below the melting temperature of the UHMWPE, xerogels will be drawn uniaxially. This will result in the transformation of the UHMWPE xerogels’ structure from a lamellar to a highly oriented state, i.e., under uniaxial tension, an extended-chain structure appears locally in the strain-hardening stage [47,48]. In addition, by applying additional drawing and increasing the drawing temperature, a fibrillar structure with a high oriented state will be achieved, i.e., the UHMWPE macromolecules will slip easily, and the distance between the extended chains will decrease, so the extension of the UHMWPE chains will be significant. Therefore, to investigate the influence of the GNP/PANI presence and its concentration on the drawing process and the influence of the drawing temperature on the final draw ratio values of the UHMWPE/PE-wax/GNP/PANI films, a multi-stage thermal orientation process was carried out using two thermal orientation regimes (Figure 2). For carrying out the thermal orientation process, a special laboratory device consisting of stepping motors and a system of sheaves was used to pass the UHMWPE/PE-wax/GNP/PANI films through a bath of silicone oil and draw them. The oil temperature was stable within ±0.1 °C precision. The multi-stage thermal orientation process for the UHMWPE films was carried out stepwise at various temperature values ranging from 120 °C to 140 °C in the first thermal orientation regime and from 120 °C to 135 °C in the second thermal orientation regime. In each stage of the thermal orientation process, each UHMWPE/PE-wax/GNP/PANI composite had a different range of a draw ratio value at different levels of temperature. Table 1 shows the draw temperatures, and the range of the achieved draw ratio for each polymer composite at each drawing stage for two thermal orientation regimes. Figure 2 presents the main stages of the oriented film preparation:

- The preparation of UHMWPE xerogels by extrusion;
- Pre-rolling of UHMWPE xerogels at a temperature of 110 °C until a deformation of 100%;
- Multi-stage thermal orientation process using uniaxial tension mode.

### 2.3. Testing Procedures

The tensile strength of the oriented UHMWPE/PE-wax/GNP/PANI films was measured using the Zwick/Roell Z020 universal testing machine (Zwick Roell Group, Ulm, Germany) according to ASTM D882-10 at a 10 mm/min loading rate. Five samples were measured for each UHMWPE composite, at least. Before carrying out the test, the surfaces of the films were cleaned with acetone to remove the silicone oil remaining from the drawing step. Since the UHMWPE films have a very low coefficient of friction, they may slip out of the grips of the testing machine during the test. An increase in grip clamping pressure may cause a premature failure in the grip area. Therefore, prior to the mechanical tests, both ends of the film were glued to 60 × 50 mm thin cardboards. The tests of the films were carried out using clamping jaws with thin notches to ensure their reliable fastening for the films without creating local stress concentrations in the capture areas. Slips of the samples during the test were not observed.

The structure of the UHMWPE/PE-wax/GNP/PANI xerogels and films was studied using a scanning electron microscope (JEOL JSM-6610LV) at an accelerating voltage of 20 kV. To avoid charge accumulation, the polymer surface was coated with a platinum layer with a thickness of 10–20 nm (magnetron deposition equipment JFC-1600, JEOL Ltd., Tokyo, Japan, was used). The lateral surface of the oriented structure of the UHMWPE/PE-wax/GNP/PANI films was obtained by the tearing mechanism in the direction of the orientation. The lateral surface of such samples was studied by SEM. For each composition, at least 10 samples were studied. To calculate the total cavitation area from SEM images, the “Image J” program (Fiji) was used. Using the “threshold” function to edit the surface of SEM images of oriented UHMWPE films made it possible to calculate the total cavitation area in percent. To calculate the average pore size and fibril lengths of highly oriented films, the “measure” function was used in the “Image J” program after setting the correct SEM image scale and determining the required length or area.

Crystallinity and melting temperature of the films were determined by differential scanning calorimetry (DSC) (NETZSCH DSC 204 F1, NETZSCH Group, Selb, Germany) at a heating rate of 10 °C min^−1^ from 35 to 180 °C in an argon atmosphere according to ASTM D 3417-83. At least, 3 samples for each UHMWPE/PE-wax/GNP/PANI composite were analyzed. The relative degree of crystallinity was calculated as the ratio of the experimental sample melting enthalpy to the completely crystallized polyethylene melting enthalpy, which is equal to 293 J/g [49]. The key parameters for DSC curve processing were T_m_^onset^ (the onset of the melting peak), T_m_ (the melting temperature peak), and T_m_^end^ (the end of the melting peak).

Dynamic mechanical analysis (DMA) was carried out using DMA Q800 (TA Instruments, USA) to evaluate the appearance of the rubbery plateau of the elastic modulus for UHMWPE xerogels [50]. The samples for DMA tests based on UHMWPE xerogels had a rectangular shape with dimensions of 40 × 2 × 0.2 mm. The prepared samples were heated from 30 °C to 180 °C at a constant rate of 2 °C/min. During heating, the samples were subjected to deformation of 0.05% and a frequency of 1 Hz.

**Figure 3:**
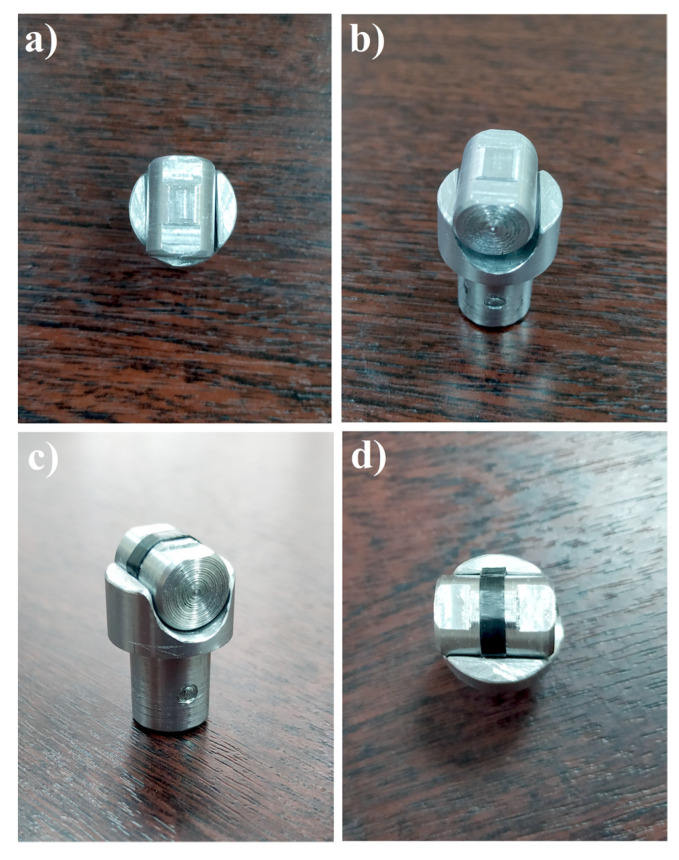
Pin form in tribological tests: (**a**,**b**) pin form without sample, (**c**,**d**) pin form with UHMWPE films samples.

Tribometer—CETR—UMT—3 (Bruker Corporation, Karlsruhe, Germany) was used to carry out the tribological tests in a dry friction mode according to ASTM G 99–95a. With a normal loading force of 30 N and a linear velocity of 1 m/s, a friction pair (pin on disk) was applied. The tribological characteristics were determined after traversing the path (L) of 21.6 km. A stainless-steel counter-body of 440C with a diameter of 62 mm was used. Diamond lapping paste with grits of 40–60 microns was used before each test to polish the counter-body. Table 2 shows the profile roughness parameters of the polished counter-body. Film samples with a length of 20 mm and a width of 2.5–4 mm were fixed to a metallic cylinder with a diameter of 20 mm, as shown in Figure 3.

**Figure 4:**
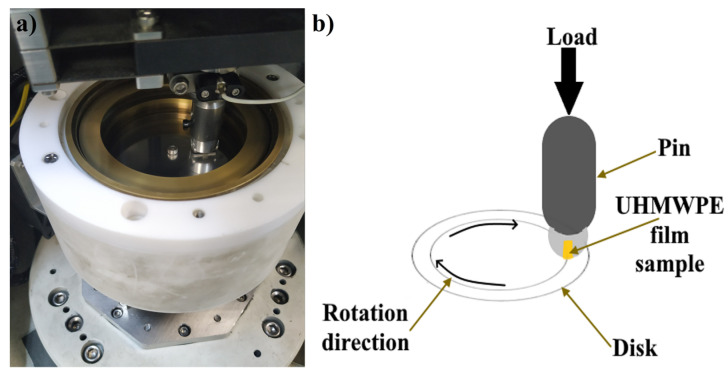
Scheme of the tribological tests: (**a**) the working place of tribometer, (**b**) illustrated draw of the tribological test.

In general, isotropic UHMWPE has a low COF value, and since this value has a considerable dependence on the applied load values [51], an increase in the applied load will lead to an increase in the COF value. Therefore, the contact pressure, which applies to the UHMWPE film samples, is considered high and equals about 4 MPa, depending on the film’s width. To maintain constant contact pressure throughout the test, a rectangle contact area was used, as shown in Figure 3a,b, to ensure stable fixation of the UHMWPE films throughout the experiment and the possibility of fixation to the counter-body. The metallic cylinder with the fixed sample was pressed against the rotating steel counter-body, as shown in Figure 4. Samples were tested in a parallel direction to the UHMWPE chain orientation. Each UHMWPE film was tested with at least three samples.

The tribological property values were recorded after 6 h of friction (21.6 km) and under a load of 30 N. Linear wear intensity (I_lW_) was calculated using Equation (1) as follows:

(1) $$I_{\mathrm{lw}}=\frac{\Delta Z}{L^{}\cdot {\;S}_{f}}$$

where: ∆Z—change in sample height after the test, L—friction path, S_f_—initial sample surface area.

## 3. Results and Discussion

As it can be seen in Table 3 and Table 4, with the increase in GNP/PANI content, the mechanical properties of oriented UHMWPE films obtained by both thermal orientation regimes decreased. For the oriented UHMWPE/PE-wax/GNP/PANI films obtained by the first thermal orientation regime, the best tensile strength value was 1080 MPa, which was obtained at a GNP/PANI content of 0.01 wt. %; the best Young’s modulus value was 33.8 GPa, which was obtained at a GNP/PANI content of 4.0 wt. %; and the best work of fracture value was 40.24 MJ/m^3^, which was obtained at a GNP/PANI content of 1.0 wt. %. Whereas, for the UHMWPE/PE-wax/GNP/PANI films obtained by the second thermal orientation regime, the best mechanical properties were obtained at a GNP/PANI content of 2.0 wt. %. The UHMWPE/1.0 wt. % PE-wax/2.0 wt. % GNP/PANI films had a tensile strength value of 1000 MPa, a Young’s modulus value of 32.1 GPa, and a work of fracture value of 49.24 MJ/m^3^. This decrease in the mechanical properties of oriented UHMWPE films is associated with the intensification of the cavitation process. However, it should be noted that the tensile strength values of the UHMWPE/PE-wax/GNP/PANI films for each GNP/PANI content were very similar for both used thermal regimes, whereas the Young’s modulus values of these films obtained by the first thermal regime were significantly higher than the ones obtained by the second thermal regime. These high Young’s modulus values are related to the higher draw ratio values for the films obtained by the first thermal regime.

**Figure 5:**
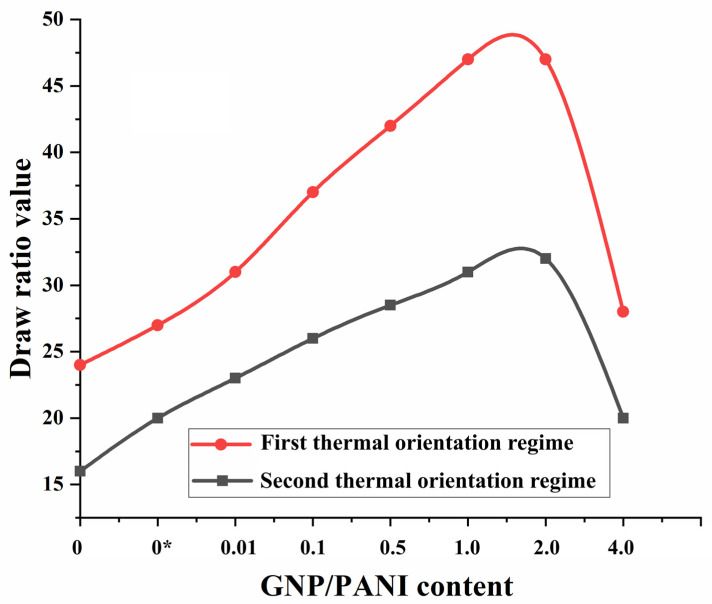
The effect of the addition of GNP/PANI on the values of the draw ratio of the UHMWPE films. (0*)—UHMWPE/1.0 wt. % PE-wax without GNP/PANI.

It was also found that the increase in the content of GNP/PANI up to 2.0 wt. % leads to an almost two-fold increase in the maximum draw ratio of UHMWPE films in comparison with oriented-virgin UHMWPE and UHMWPE/PE-wax films for both thermal orientation regimes (Figure 5). During solid-phase mixing in the planetary ball mill, GNP/PANI are distributed over the surface of the UHMWPE particles, and each UHMWPE particle can then give a separate microfibril during the thermal orientation process. Due to the presence of GPN/PANI on the surface of UHMWPE particles between fibrils during the thermal orientation process, interfibrillar sliding with intensive fibrillation can occur, which increases the final draw ratio of the films, but is not accompanied by an increase in strength properties. Moreover, GNP/PANI agglomerates were not observed in the UHMWPE matrix (Figure S5).

**Figure 6:**
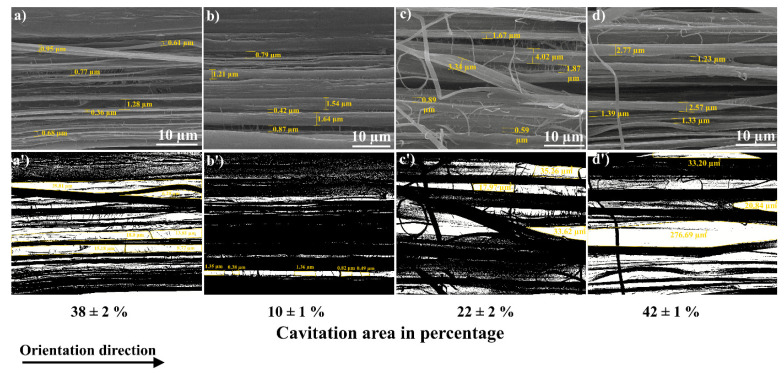
SEM micrographs of fibrillar structure for UHMWPE films obtained by first thermal orientation regime: (**a**)—virgin UHMWPE (average pore size is 1.2 ± 0.2 µm), (**b**)—UHMWPE/1.0% PE-wax (average pore size is 0.8 ± 0.2 µm), (**c**)—UHMWPE/1.0 wt. % PE-wax/0.01 wt. % GNP/PAN (average pore size is 3.4 ± 0.5 µm), (**d**)—UHMWPE/1.0 wt. % PE-wax/2.0 wt. % GNP/PAN (average pore size is 3.7 ± 0.4 µm); (**a’**–**d’**)—modified SEM images using the image J program.

Moreover, for the films prepared based on the first thermal regime, it was found that the addition of GNP/PANI resulted in an increase in total cavitation area with increasing GNP/PANI content compared to the oriented-virgin UHMWPE and UHMWPE/PE-wax films, resulting in an increase in the maximum obtained draw ratio. An increase in the total cavitation area by 120–320% with an increase in the GNP/PANI content in comparison with oriented-virgin UHMWPE and UHMWPE/PE-wax films is shown in Figure 6 (also Figures S6–S8 and S10). Moreover, the addition of GNP/PANI leads to a significant increase in pore sizes by 300–400% compared to oriented-virgin UHMWPE films, and this increase in pore sizes has occurred by increasing the GNP/PANI content and by increasing the draw ratio.

**Figure 7:**
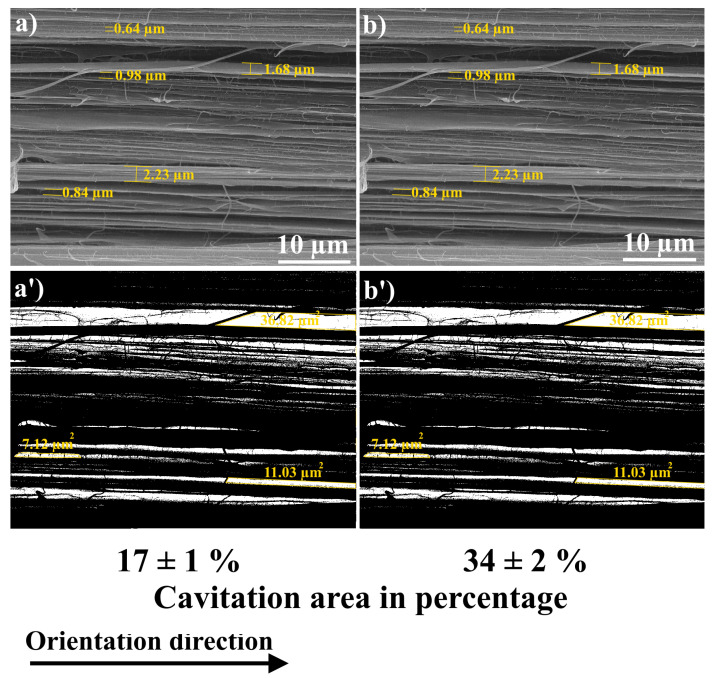
SEM micrographs of fibrillar structure for UHMWPE films obtained by second thermal orientation regime: (**a**)—UHMWPE/1.0 wt. % PE-wax/0.01 wt. % GNP/PAN (average pore size is 1.8 ± 0.2 µm), (**b**)—UHMWPE/1.0 wt. % PE-wax/2.0 wt. % GNP/PAN (average pore size is 2.2 ± 0.3 µm); (**a’**,**b’**)—modified SEM images using the image J program.

As it can be seen in Table 3 and Table 4, the virgin-oriented UHNWPE and UHMWPE/PE-wax films had almost the same draw ratio values for both applied thermal regimes. Therefore, in order to better understand the role of the draw ratio on the structure and cavitation process of the UHMWPE/PE-wax/GNP/PANI, the films obtained by the second thermal regime (with a lower draw ratio in comparison with the films obtained by the first regime) with GNP/PANI contents of 0.01 and 2.0 wt. % were investigated and compared with the films that have the same GNP/PANI contents. As it can be seen in Figure 7 (also Figures S9 and S11), the cavitation area and pore size were smaller for the UHMWPE/PE-wax/GNP/PANI films obtained by the second thermal regime in comparison with the films obtained by the first thermal regime. These results are related to the lower draw ratio of the films that were prepared based on the second thermal regime, which means a lower fibrillation process in comparison with the films obtained by the first thermal regime. 

The intensification of cavitation processes upon the addition of the GNP/PANI to the UHMWPE matrix is associated with a deterioration in the diffusion of macromolecules between UHMWPE particles during the preparation of the UHMWPE xerogels. In the oriented UHMWPE films filled with GNP/PANI, the bond between microfibrils will be worse compared to that in oriented virgin UHMWPE films. When the tensile stresses are applied in the orientation process, the microfibrils in the transverse direction are more easily separated from each other, which leads to the intensification of cavitation processes.

**Figure 8:**
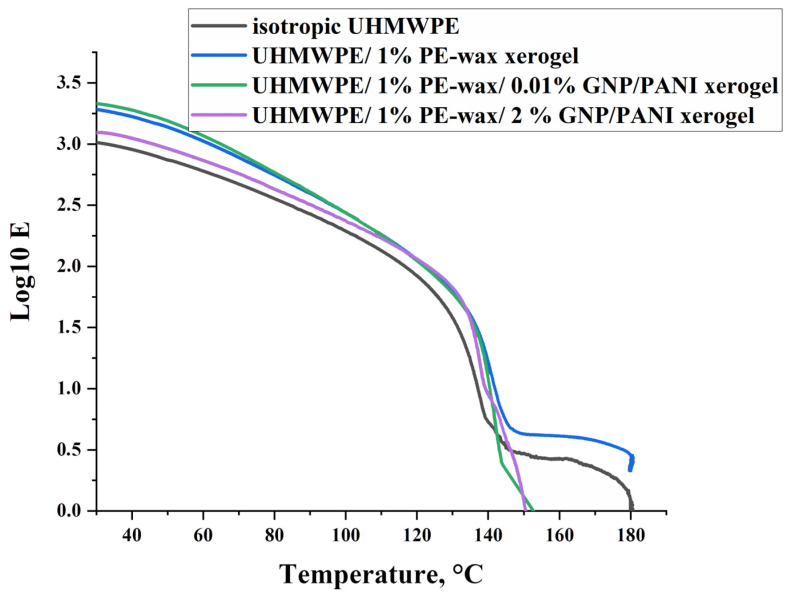
Curves of Young’s modulus plateau for UHMWPE samples measured under dynamic loading.

The appearance of an elastic modulus plateau at temperatures above the polymer’s melting point is associated with a high density of physical links (entanglements or crosslinks) between polymer macromolecules, which can retain elastic properties in the material after melting the crystalline phase [53]. Sliding of UHMWPE macromolecules inside the material at the GNP/PANI interfaces is confirmed by DMA studies, in which the appearance of the plateau of the elastic modulus of UHMWPE xerogels in the melting temperature region, is related to the presence of the entanglements (Figure 8). For isotropic UHMWPE and UHMWPE/1%PE-wax xerogels at a temperature of 150 °C, the elastic modulus reaches a plateau, which is associated with a high density of physical entanglements between UHMWPE macromolecules, which retain the elastic properties in the material after melting of the crystalline phase [53,54]. For the UHMWPE xerogels with GNP/PANI contents of 0.01 and 2.0 wt. %, the modulus of elasticity drops to zero at 150 °C. The reason for this may be the intense internal slippage of the material over surfaces saturated with GNP/PANI and containing a low density of transverse (tie) macromolecules.

Table 5 and Table 6 show the DSC test results for the UHMWPE/1.0 wt. % PE-wax/GNP/PANI films obtained by both thermal orientation regimes. As it can be seen in these tables, the obtained UHMWPE/1.0 wt. % PE-wax/GNP/PANI films had high crystallinity values because of the orientation process of the UHMWPE macromolecules and the transformation of the lamellae to fibrillar structure (recrystallization), which in turn leads to a reduction of the defects of the crystalline phase [55]. It is related to the presence of the PE-wax as an intermolecular lubricant, which promotes recrystallization processes as a result of the greater mobility of the UHMWPE macromolecules. For the UHMWPE/1.0 wt. % PE-wax/GNP/PANI films obtained by the first thermal orientation regime, the influence of the GNP/PANI addition on the melting temperature was not observed; but, for the films obtained by the second one, the melting temperature values of the UHMWPE/1.0 wt. % PE-wax/GNP/PANI films were increased.

The behavior of the friction coefficient is determined by the adhesive and deformation components. The adhesive component is associated with intermolecular interactions on friction surfaces, which can be different in nature. Intermolecular interactions exist not only in places of direct contact between rubbing surfaces, but also at some distances where there is no direct contact. The deformation component arises because of the bulk deformation of the friction surfaces, which leads to the penetration of the surface of a more rigid material into a softer one, an increase in the contact friction area, and an increase in the roughness of the friction surfaces. The embedded element, moving in the tangential direction, either deforms the surface of a softer material, or cuts it off. The role of these two types of friction coefficient components depends on the conditions under which friction occurs. For example, an increase in the applied load leads to an increase in plastic deformation, which, in turn, leads to an increase in the deformation component. On the other hand, an increase in load leads to a decrease in the adhesive component while maintaining surface roughness because of the increase in shear stresses that destroy molecular bonds. Thus, considering the role of each component in a particular friction unit, it is possible to minimize the values of the friction coefficient [56].

**Figure 9:**
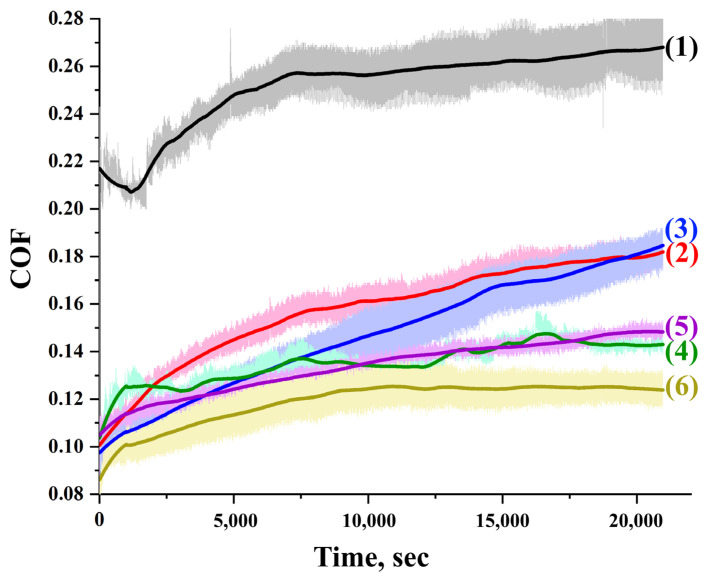
Coefficient of friction curves for isotropic and oriented UHMWPE films with different GNP/PANI contents. (1) isotropic UHMWPE, (2) oriented UHMWPE film, (3) oriented UHMWPE/1.0 wt. % PE-wax film, (4) oriented UHMWPE/1.0 wt. % PE-wax/0.01 wt. % GNP/PANI film, (5) oriented UHMWPE/1 wt. % PE-wax/0.5 wt. % GNP/PANI film, (6) oriented UHMWPE/1 wt. % PE-wax/2 wt. % GNP/PANI film. All samples were obtained by first thermal orientation regime.

**Figure 10:**
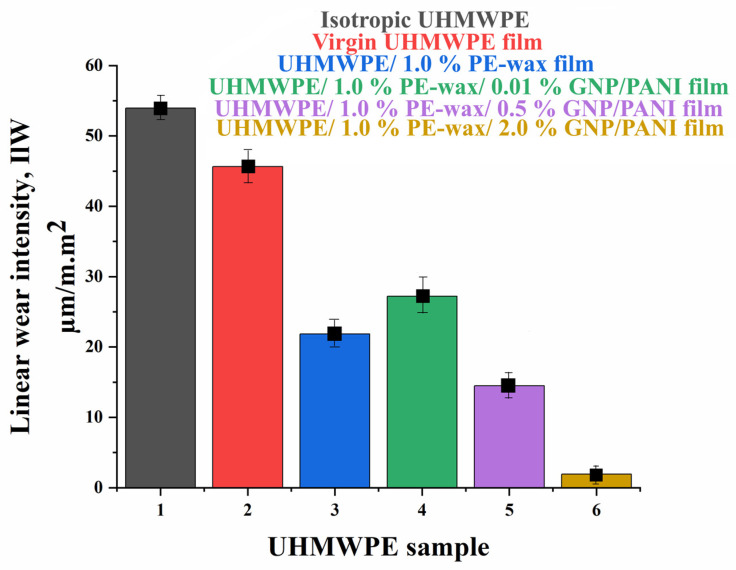
Linear wear intensity for UHMWPE samples obtained at drawing temperature of 140 °C, where (1) isotropic UHMWPE, (2) virgin-oriented UHMWPE, (3) oriented UHMWPE/1.0% PE-wax, (4) oriented UHMWPE/1.0% PE-wax/0.01% GNP/PANI, (5) oriented UHMWPE/1.0% PE-wax/0.5% GNP/PANI, and (6) oriented UHMWPE/1.0% PE-wax/2.0% GNP/PANI. All samples were obtained by first thermal orientation regime.

Figure 9 and Figure 10 and Table 7 present the results of tribological tests demonstrating the effect of draw ratio, PE-wax, and GPN/PANI content on friction coefficient and wear resistance for the films obtained by the first thermal orientation regime. The change in the friction coefficient for all tested materials occurred in two stages, as follows: Stage 1–increasing the coefficient of friction at the beginning of the test as the surface adapts to wear; Stage 2–the friction coefficient reaches a plateau due to the formation of a tribo-layer, which helps to reduce friction, which in turn reduces abrasive wear and the deformation component of the friction coefficient [57,58].

For isotropic UHMWPE, when a high load (30 N) was applied, the friction coefficient tended to gradually increase and stabilize at 0.269. The appearance of the orientation of UHMWPE macromolecules leads to a significant decrease in the friction coefficient from 0.269 for isotropic UHMWPE to 0.179 for oriented UHMWPE. The decrease in the friction coefficient is associated with an increase in the mechanical properties of UHMWPE because of the thermal orientation hardening process, which increases the rigidity of the material and reduces the deformation component of the friction coefficient (reducing the penetration of the counter-body into the surface of the material under test) [21,22,23,24]. The addition of the PE-wax contributes to an additional reduction in the initial moment of the coefficient of friction due to its action as a lubricant and an increase in the mechanical properties of the UHMWPE/PE-wax films. Increasing the time of the friction test led to an increase in the coefficient of friction up to 0.171 (near the COF value of the oriented virgin UHMWPE films), due to a change in the surface roughness of the UHMWPE/PE-wax films. The addition of GNP/PANI antifriction particles to the UHMWPE matrix contributed to an additional reduction in the friction coefficient and an increase in wear resistance (Figure 9 and Figure 10, and Table 7) due to the lubricating effect of carbon materials. GNP/PANI reduce the coefficient of friction from 0.179 to 0.122 for the films with a content of GNP/PANI of 2.0 wt. % [27,28,29].

From Figure 10, the appearance of orientation in the UHMWPE macromolecules leads to a slight decrease in wear in comparison with the isotropic UHMWPE. With the addition of PE-wax, wear is reduced from 45.63 µm/m.m^2^ for the oriented virgin UHMWPE films to 21.83 µm/m.m^2^ for UHMWPE/PE wax films, by improving the mechanical properties and increasing the material stiffness as a result of the thermal orientation stretching process. Because GNPs are highly abrasion resistant, increasing the GNP/PANI content results in an additional reduction in wear values. A minimum wear value of 1.92 µm/m.m^2^ was obtained for oriented UHMWPE/PE-wax/GNP/PANI films with a GNP/PANI content of 2.0 wt. % obtained by the first thermal orientation regime. From Table 7, the content of GNP/PANI greater than 2 wt. % did not have a positive effect on the values of the coefficient of friction and wear resistance due to a decrease in the maximum draw ratio of the films [27,59,60].

**Figure 11:**
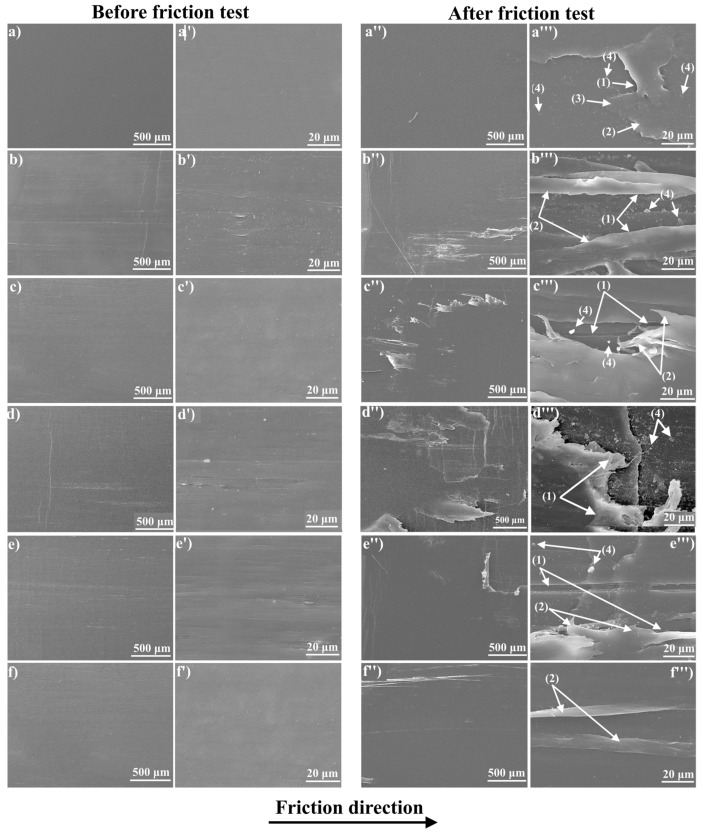
SEM micrographs of the UHMWPE films before and after friction tests, (**a,a’,a’’,a’’’**) isotropic UHMWPE, (**b,b’,b’’,b’’’**) virgin oriented UHMWPE, (**c,c’,c’’,c’’’**) oriented UHMWPE/1.0% PE-wax, (**d,d’,d’’,d’’’**) oriented UHMWPE/1.0% PE-wax/0.01% GNP/PANI, (**e,e’,e’’,e’’’**) oriented UHMWPE/1.0% PE-wax/0.5% GNP/PANI, and (**f,f’,f’’,f’’’**) oriented UHMWPE/1.0% PE-wax/2.0% GNP/PANI; (1) abrasive wear, (2) delamination (waves), (3) elongated polymer particles, and (4) wear particles. a–f: samples before friction with a magnification of 500 µm, (**a’**–**f’**): samples before friction with a magnification of 20 µm, (**a’’**–**f’’**): samples after friction with a magnification of 500 µm, (**a’’’**–**f’’’**): samples after friction with a magnification of 20 µm. All samples were obtained by first thermal orientation regime.

Figure 11 shows the SEM micrographs of the friction surfaces of UHMWPE samples before and after tribological tests. The movement of the counter-body in one direction during tribological tests leads to the appearance of an oriented structure on the surface of isotropic UHMWPE. On the other hand, on the surfaces of the oriented UHMWPE and UHMWPE/PE-wax films, traces of strong abrasive wear and waves oriented perpendicular to the direction of friction were found. During long-term operation of oriented UHMWPE and UHMWPE/PE-wax films, fatigue wear mechanisms will occur. Microstructural changes recognized with frictional loading are a precursor to the formation of fatigue microcracks on the friction surface (Figure 11). This process is characterized by the formation of cracks on the polymer surface, which cause delamination and the formation of large wear particles [12,61]. Moreover, it was found that the presence of GNP/PANI leads to a reduction in fatigue and a significant reduction in cracks. The positive effect of GNP/PANI can be caused by the uniform distribution of GNP/PANI in the UHMWPE matrix and the formation of a tribolayer with a lubricating effect on the surface of oriented UHMWPE films. As can be seen in Figure 11, minimal damage to the friction surface occurs for UHMWPE/PE-wax/GNP/PANI films at GNP/PANI content of 2.0 wt. %.

**Figure 12:**
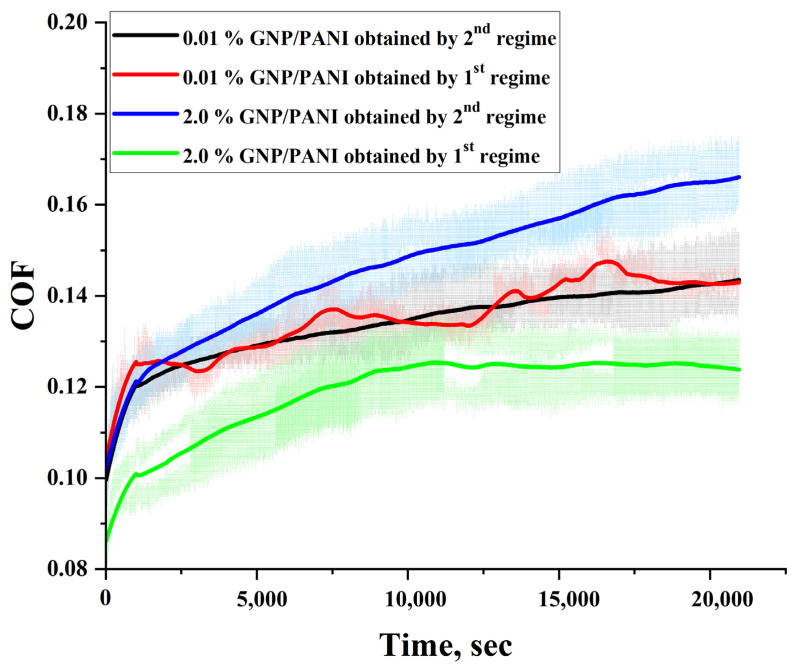
Coefficient of friction curves for UHMWPE/1.0% PE-wax/GNP/PANI films obtained by second thermal orientation regime in comparison with the films obtained by the first regime.

**Figure 13:**
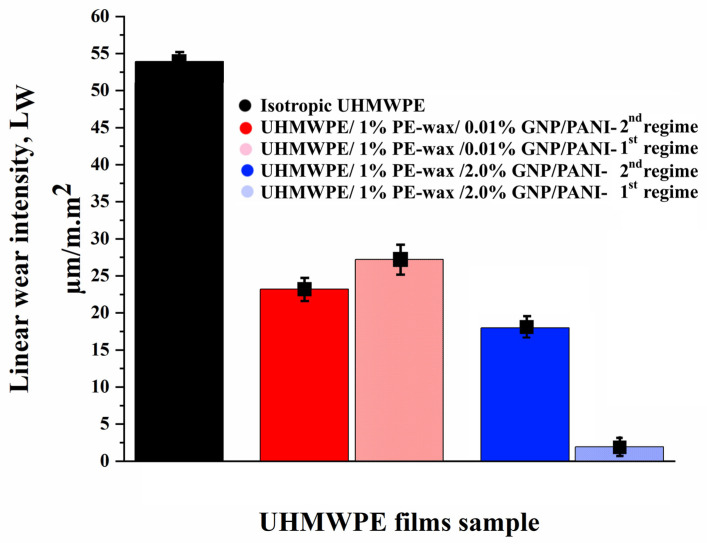
Linear wear intensity for UHMWPE/1.0% PE-wax/GNP/PANI films obtained by second thermal orientation regime in comparison with the films obtained by the second regime.

Once again, in order to better understand the role of the draw ratio on the tribological properties of the UHMWPE/PE-wax/GNP/PANI, the films obtained by the second thermal regime with GNP/PANI contents of 0.01 and 2.0 wt. % were investigated and compared with the films that have the same GNP/PANI contents. As can be seen in Table 8 and Figure 12 and Figure 13, for the UHMWPE/1.0% PE-wax/GNP/PANI films with a GNP/PANI content of 0.01 wt. % obtained by the first and second thermal orientation regimes, the COF and linear wear intensity values were not changed by increasing the draw ratio (DR) values. Since the mechanical properties and the crystallinity of these films are very similar, this small amount of GNP/PANI leads to an improvement in the DR values, but does not improve the tribological properties. On the other hand, for the UHMWPE/1.0% PE-wax/GNP/PANI films with a GNP/PANI content of 2.0 wt. %, both COF and linear wear intensity values were significantly improved when the DR value was increased. Because the mechanical properties and crystallinity of these films are so similar, this improvement can be attributed to the high DR value and sufficiently high GNP or PANI content.

## 4. Conclusions

The influence of the GNP/PANI addition to the UHMWPE matrix on the structure, formation of voids (cavitation process), mechanical, and tribological properties was studied for the highly oriented UHMWPE films. A thermal orientation process was used to prepare highly oriented UHMWPE films. The findings indicated that the increase in the content of GNP/PANI up to 2.0 wt. % had led to an increase in the maximum draw ratio of UHMWPE films by approximately two times in comparison with the oriented virgin UHMWPE films. The explanation could be as follows. The interfibrillar sliding during the intensive fibrillation process during the thermal orientation process led to an intensification of the cavitation processes, which in turn led to a decrease in the mechanical properties of the highly oriented UHMWPE/PE-wax/GNP/PANI films. In comparison with the oriented-virgin UHMWPE films, SEM micrographs revealed an increase in total cavitation area of 120–320% and pore size of 300–400% by the increase in GNP/PANI content. Based on DMA tests for UHMWPE xerogels, the appearance of the elastic modulus plateau in the melting temperature region was confirmed, indicating the sliding of the UHMWPE macromolecules inside the material at the GNP/PANI interfaces, which could be related to the intense internal slippage in the material over surfaces saturated with GNP/PANI and containing a low density of transverse (tie) macromolecules. 

The influence of each component in the oriented UHMWPE films can be distinguished based on the results of the tribological tests as follows:

- PE-wax functions as a lubricant with a low molecular weight.
- In comparison to isotropic UHMWPE, the thermal orientation drawing process increases mechanical properties, which increases material rigidity and reduces the deformation component of the friction coefficient (reducing counter-body penetration into the surface of the material being tested).
- In the process of the friction in the surface layers of the polymer matrix involved in the friction, an intense movement of the macromolecules is observed, aimed at reorienting the polymer chains in the direction of the friction. The reduction of the shear deformations for the oriented films is related to the fact that the reorientation of the UHMWPE macromolecules is not required, which in turn leads to a decrease in the coefficient of friction.
- The fibrillar structure of the oriented films contributes to an increase in the resistance to fatigue strength during friction.
- GNP has a high resistance to abrasive wear, which means that incorporating GNP/PANI into the polymer matrix reduces overall composite wear.

Based on the obtained UHMWPE films, the best ones are UHMWPE/PE-wax/GNP/PANI films with a GNP/PANI content of 2.0 wt. %. They have excellent mechanical and tribological properties in terms of tensile strength of 836 MPa, Young’s modulus of 35.8 GPa, and COF of 0.122. These films have a high crystallinity of 87%, but their cavitation area is also high (42%), and their average pore size is 3.7 ± 0.4 µm, which explains the decrease in their tensile strength in comparison with UHMWPE/PE-wax films.

Finally, these highly oriented UHMWPE/PE-wax/GNP/PANI films with excellent mechanical and tribological properties are prepared from cheap large-scale grades of UHMWPE. Therefore, they are considered the best choice for both biomedical and industrial applications, especially tribological applications (sliding bearings). They can be widely used as materials that form the friction surface, such as coatings for plain bearings, various guides, and rollers operating under hard, dry friction conditions.

## Figures and Tables

**Table 1:** Thermal orientation regimes and achieved values of the drawing ratio at each stage of thermal orientation process for UHMWPE composites filled with PE-wax and GNP/PANI.

Type of Thermal Orientation Regime	Temperature, °C	Draw Ratio, λ
First thermal orientation regime	120	5–6
	130	9–11
	135	14–16
	140	19–47
Second thermal orientation regime	120	5–6
	130	9–11
	135	16–32

**Table 2:** Profile roughness parameters of used counter-body in tribological tests.

R_a_, nm	R_q_, nm	R_z_, µm	R_t_, µm
72.84 ± 1.81	96.15 ± 2.57	1.02 ± 0.09	1.35 ± 0.28

R_a_—roughness average, R_q_—root mean square roughness (RMS), R_z_—average maximum height of profile, R_t_—maximum height of profile.

**Table 3:** Mechanical tensile properties of the UHMWPE/PE-wax/GNP/PANI films with different GNP/PANI content obtained by first thermal orientation regime (±the standard deviation).

Material	GNP/PANI Content, wt. %	Final DR	Young’s Modulus, GPa	Tensile Strength, MPa	Elongation, %	Work of Fracture, MJ/m^3^
Oriented UHMWPE films	0	19	18.2 ± 1.3	720 ± 32	5.1 ± 0.4	10.1 ± 0.2
UHMWPE/1.0% PE-Wax	0	23	31.6 ± 2.0	1320 ± 31	7.35 ± 1.4	106.6 ± 10.1
UHMWPE/1.0%PE-Wax/GNP/PANI films	0.01	31	31.4 ± 2.8	1080 ± 70	5.6 ± 0.8	39.3 ± 7.3
	0.1	32	25.1 ± 0.6	623 ± 43	5.4 ± 0.9	39.4 ± 2.6
	0.5	45	31.7 ± 2.3	826 ± 33	6.2 ± 1.0	37.3 ± 4.1
	1.0	46	32.4 ± 1.1	716 ± 15	8.9 ± 0.4	40.2 ± 0.5
	2.0	47	35.8 ± 3.3	836 ± 88	6.6 ± 0.9	40.3 ± 11.6
	4.0	28	33.8 ± 3.2	688 ± 21	3.5 ± 0.6	27.9 ± 6.1

Isotropic GUR 4120 has yield strength of ≥17 MPa, Young’s modulus of 720 MPa, and nominal elongation at break of ≥50 % [52].

**Table 4:** Mechanical tensile properties of the UHMWPE/PE-wax/GNP/PANI films with different GNP/PANI content obtained by second thermal orientation regime (±the standard deviation).

Material	GNP/PANI Content, wt. %	Final DR	Young’s Modulus, GPa	Tensile Strength, MPa	Elongation, %	Work of Fracture, MJ/m^3^
Oriented UHMWPE films	0	16	7.6 ± 1.7	482 ± 7	7.5 ± 2.1	10.39 ± 4.45
UHMWPE/1.0% PE-Wax	0	20	16.7 ± 3.1	738 ± 60	4.0 ± 1.5	9.03 ± 1.70
UHMWPE/1.0%PE-Wax/GNP/PANI films	0.01	23	27.0 ± 4.0	898 ± 64	4.6 ± 0.4	11.17 ± 1.49
	0.1	24	12.0 ± 1.6	571 ± 79	6.2 ± 0.8	10.03 ± 1.87
	0.5	28	26.8 ± 2.3	836 ± 28	4.1 ± 0.8	9.5 ± 1.47
	1.0	30	25.6 ± 0.2	759 ± 90	5.6 ± 0.8	7.67 ± 1.87
	2.0	32	32.1 ± 1.8	1000 ± 13	7.9 ± 1.3	49.24 ± 5.3
	4.0	20	14.8 ± 1.2	519 ± 75	4.1 ± 0.6	5.85 ± 1.38

**Table 5:** DSC test results for the UHMWPE/1.0 wt. % PE-wax/GNP/PANI films obtained by first thermal orientation regime (±the standard deviation).

Material	GNP/PANI Content, wt. %	T_m_^onset^, °C	T_m_, °C	T_m_^end^, °C	Crystallinity, %
Oriented UHMWPE	0	139.5	142.1	144.3	77 ± 2
UHMWPE/1.0 wt. % PE-wax	0	138.9	142.4	145.7	87 ± 3
UHMWPE/1.0 wt. % PE- wax/GNP/PANI	0.01	141.6	144.1	145.7	87 ± 3
	0.1	137.5	142.8	144.6	81 ± 2
	0.5	137.8	142.9	144.7	80 ± 2
	1.0	139.2	143.1	145.0	86 ± 2
	2.0	140.8	143.6	145.7	87 ± 2

**Table 6:** DSC test results for the UHMWPE/1.0 wt. % PE-wax/GNP/PANI films obtained by second thermal orientation regime (±the standard deviation).

Material	GNP/PANI Content, wt. %	T_m_^onset^, °C	T_m_, °C	T_m_^end^, °C	Crystallinity, %
Oriented UHMWPE	0	130.3	140.3	147.8	73 ± 2
UHMWPE/1.0 wt. % PE-wax	0	136.5	142.4	144.4	81 ± 2
UHMWPE/1.0 wt. % PE- wax/GNP/PANI	0.01	139.2	143.0	145.1	91 ± 3
	2.0	141.6	143.7	145.5	87 ± 3

**Table 7:** Tribological properties of isotropic UHMWPE, virgin-oriented UHMWPE film, oriented UHMWPE/1.0% PE-wax films, and oriented UHMWPE/1.0% PE-wax/GNP/PANI films obtained by first thermal orientation regime (±the standard deviation).

Material	GNP/PANI Content, wt. %	Draw Ratio	COF	Linear Wear Intensity, I_lW_ µm/m.m^2^
Isotropic virgin UHMWPE	0	-	0.269 ± 0.015	53.94 ± 0.27
Oriented Virgin UHMWPE films	0	19	0.179 ± 0.013	45.63 ± 0.68
Oriented UHMWPE/1% PE-wax films	0	23	0.171 ± 0.005	21.83 ± 0.42
Oriented UHMWPE/1% PE-wax/GNP/PANI films	0.01	31	0.150 ± 0.002	27.20 ± 0.76
	0.1	32	0.167 ± 0.008	31.32 ± 0.39
	0.5	45	0.144 ± 0.009	14.49 ± 0.45
	1.0	46	0.168 ± 0.004	11.35 ± 0.35
	2.0	47	0.122 ± 0.002	1.92 ± 0.08
	4.0	28	0.187 ±0.013	16.59 ± 0.05

**Table 8:** Tribological properties of isotropic UHMWPE, virgin-oriented UHMWPE film, oriented UHMWPE/1.0% PE-wax films, and oriented UHMWPE/1.0% PE-wax/GNP/PANI films obtained by second thermal orientation regime (±the standard deviation).

Material	GNP/PANI Content, wt. %	Draw Ratio	COF	Linear Wear Intensity, I_lw_, µm/m.m^2^
Oriented UHMWPE/1% PE-wax/0.01% GNP/PANI	0.01	23	0.141 ± 0.002	23.3 ± 0.49
Oriented UHMWPE/1% PE-wax/2% GNP/PANI	2.0	32	0.157 ± 0.003	18.00 ± 0.54

## Data Availability

The data that support the findings of this study are openly available in the Russian Science Foundation at [https://rscf.ru/en/project/22-73-00136/], reference number 22-73-00136. The authors confirm that the data supporting the findings of this study are available within the article andits supplementary materials.

## Supplementary Materials

The following supporting information can be downloaded at: https://www.mdpi.com/article/10.3390/polym15030758/s1, Figure S1: The scheme of oxidative polymerization of aniline; Figure S2: SEM and TEM micrographs of GNP microstructure; Figure S3: SEM micrograph of the unmodified GNP and modified GNP by PANI; Figure S4: Raman spectra of unmodified GNP and modified GNP by PANI; Figure S5: SEM micrographs showing typical supra-molecular structure of UHMWPE /1.0 wt. % PE-wax/GNP/PANI xerogels; Figure S6: SEM micrographs of fibrillar structure for virgin UHMWPE films obtained by first thermal orientation regime; Figure S7: SEM micrographs of fibrillar structure for UHMWPE/1.0 wt. % PE-wax films obtained by first thermal orientation regime; Figure S8: SEM micrographs of fibrillar structure for UHMWPE /1.0 wt. % PE-wax/ 0.01 wt. % GNP/PAN films obtained by first thermal orientation regime; Figure S9: SEM micrographs of fibrillar structure for UHMWPE /1.0 wt. % PE-wax/ 0.01 wt. % GNP/PAN films obtained by second thermal orientation regime; Figure S10: SEM micrographs of fibrillar structure for UHMWPE /1.0 wt. % PE-wax/ 2.0 wt. % GNP/PANI films obtained by first thermal orientation regime; Figure S11: SEM micrographs of fibrillar structure for UHMWPE /1.0 wt. % PE-wax/ 2.0 wt. % GNP/PAN films obtained by second thermal orientation regime.

## References

[1] Friedrich K., Schlarb A. (2008). Tribology of Polymeric Nanocomposites, Friction and Wear of Bulk Materials and Coatings.

[2] Sturzel M., Mihan S., Mulhaupt R. (2016). From Multisite Polymerization Catalysis to Sustainable Materials and All-Polyolefin Composites. Chem. Rev..

[3] Laska A. (2017). Comparison of conventional and crosslinked ultra high molecular weight polyethylene (UHMWPE) used in hip implant. World Sci. News.

[4] Dougherty P.S.M., Pudjoprawoto R., Higgs III C.F. (2011). An investigation of the wear mechanism leading to self-replenishing transfer films. Wear.

[5] Rhee S.H., Ludema K.C. (1978). Mechanisms of formation of polymeric transfer films. Wear.

[6] Schwartz C.J., Bahadur S. (2000). Studies on the tribological behavior and transfer film-counterface bond strength for polyphenylene sulfide filled with nanoscale alumina particles. Wear.

[7] Galetz M.C., Blaβ T., Ruckdäschel H., Sandler J.K.W., Altstädt V., Glatzel U. (2007). Carbon nanofibre-reinforced ultrahigh molecular weight polyethylene for tribological applications. J. Appl. Polym. Sci..

[8] Sreekanth P.S.R., Kanagaraj S. (2015). Influence of multi walled carbon nanotubes reinforcement and gamma irradiation on the wear behaviour of UHMWPE. Wear.

[9] Typical Properties of NYLON Curbell Plastics Acquired Nationwide Plastics in 2019. https://www.curbellplastics.com/Research-Solutions/Materials/Nylon.

[10] Typical Properties of ACETAL Curbell Plastics Acquired Nationwide Plastics in 2019. https://www.curbellplastics.com/Research-Solutions/Materials/Acetal.

[11] Budinski K.G. (1997). Resistance to particle abrasion of selected plastics. Wear.

[12] Eddoumy F., Addiego F., Dhieb H., Célis J.-P., Muller R., Toniazzo V., Ruch D. (2012). Sliding wear behaviour of oriented ultrahigh molecular weight polyethylene. Polym. Int..

[13] Li J., Guo Z., Hua M., Qin X., Wen S. (2004). Tribological characteristics of UHMWPE composite and relationship with its compressive behavior. Sci. China Ser. G Phys. Mech. Astron..

[14] Smith P., Lemstra P.J., Pijpers J.P.L., Kiel A.M. (1980). Ultra-drawing of high molecular weight polyethylene cast from solution. Colloid Polym. Sci..

[15] Kuo C.J., Lan W.L., Zhang D. (2014). Gel Spinning of Synthetic Polymer Fibres. Advances in Filament Yarn Spinning of Textiles and Polymers.

[16] Cao J., Gao X., Shen K. (2012). Morphologies and mechanical properties of high-density polyethylene induced by the addition of small amounts of both low-and high-molecular-weight polyolefin under shear stress applied by dynamic packing injection molding. J. Macromol. Sci. Part B Phys..

[17] Dayyoub T., Olifirov L.K., Chukov D.I., Kaloshkin S.D., Kolesnikov E., Nematulloev S. (2020). The Structural and Mechanical Properties of the UHMWPE Films Mixed with the PE-Wax. Materials.

[18] Jarecki L., Pecherski R.B. (2018). Kinetics of oriented crystallization of polymers in the linear stress-orientation range in the series expansion approach. Express Polym. Lett..

[19] Sanborn B., DiLeonardi A., Weerasooriya T., Dynam J. (2015). Tensile Properties of Dyneema SK76 Single Fibers at Multiple Loading Rates Using a Direct Gripping Method. J. Dyn. Behav. Mater..

[20] Dayyoub T., Maksimkin A.V., Kaloshkin S., Kolesnikov E., Chukov D., Dyachkova T.P., Gutnik I. (2019). The Structure and Mechanical Properties of the UHMWPE Films Modified by the Mixture of Graphene Nanoplates with Polyaniline. Polymers.

[21] Sambasivan S., Fischer D.A., Shen M.C., Hsu S.M. (2004). Molecular orientation of ultrahigh molecular weight polyethylene induced by various sliding motions. J. Biomed. Mater. Res. B Appl. Biomater..

[22] Dharmastiti R., Barton D.C., Fisher J., Edidin A., Kurtz S. (2001). The wear of oriented UHMWPE under isotropically rough and scratched counterface test conditions. Biomed Mater Eng..

[23] Chang N., Bellare A., Cohen R.E., Spector M. (2000). Wear behavior of bulk oriented and fiber reinforced UHMWPE. Wear.

[24] Wang A., Sun D.C., Yau S.-S., Edwards B., Sokol M., Essner A., Polineni V.K., Stark C., Dumbleton J.H. (1997). Orientation softening in the deformation and wear of ultra-high molecular weight polyethylene. Wear.

[25] Hees T., Zhong F., Koplin C., Jaeger R., Mülhaupt R. (2018). Wear resistant all-PE single-component composites via 1D nanostructure formation during melt processing. Polymer.

[26] Puértolas J.A., Kurtz S.M. (2016). UHMWPE Matrix Composites. UHMWPE Biomaterials Handbook.

[27] Lahiria D., Hec F., Thiesse M., Durygin A., Zhang C., Agarwal A. (2014). Nanotribological behavior of graphene nanoplatelet reinforced ultra-high molecular weight polyethylene composites. Tribol. Int..

[28] Tai Z., Chen Y., An Y., Yan X., Xue Q. (2012). Tribological Behavior of UHMWPE Reinforced with Graphene Oxide Nanosheets. Tribol. Lett..

[29] Bhattacharyya A., Chen S., Zhu M. (2014). Graphene reinforced ultra-high molecular weightpolyethylene with improved tensile strength and creepresistance properties. Express Polym. Lett..

[30] Revanappa S.K., Soni I., Siddalinganahalli M., Jayaprakash G.K., Flores-Moreno R., Bananakere Nanjegowda C. (2022). A Fukui Analysis of an Arginine-Modified Carbon Surface for the Electrochemical Sensing of Dopamine. Materials.

[31] Caupin F., Herbert E. (2006). Cavitation in water: A review. Comptes Rendus Phys..

[32] Hayward A.T.J. (1970). The role of stabilized gas nuclei in hydrodynamic cavitation inception. J. Phys. D.

[33] Galeski A., Koenczoel L., Piorkowska E., Baer E. (1987). Acoustic emission during polymer crystallization. Nature.

[34] Galeski A., Piorkowska E., Koenczoel L., Baer E. (1990). Acoustic emission during crystallization of polymers. J. Polym. Sci. Part B Polym. Phys..

[35] Nowacki R., Kolasinska J., Piorkowska E. (2001). Cavitation during isothermal crystallization of isotactic polypropylene. J. Appl. Polym. Sci..

[36] Nowacki R., Piorkowska E. (2007). Influence of solid particles on cavitation in poly (methylene oxide) during crystallization. J. Appl. Polym. Sci..

[37] Castagnet S., Girault S., Gacougnolle J.L., Dang P. (2000). Cavitation in strained polyvinylidene fluoride: Mechanical and X-ray experimental studies. Polymer.

[38] Lin L., Argon A.S. (1994). Structure and plastic deformation of polyethylene. J Mater. Sci..

[39] Bowden P.B., Young R.J. (1974). Deformation mechanisms in crystalline polymers. J. Mater. Sci..

[40] Rozanski A. (2010). Initiation of Cavitation during Drawing of Crystalline Polymers. Ph.D. Thesis.

[41] Pawlak A., Galeski A. (2008). Cavitation during Tensile Deformation of Polypropylene. Macromolecules.

[42] Liu Y., Truss R.W. (1994). A study of tensile yielding of isotactic polypropylene. J. Polym. Sci. Part B Polym. Phys..

[43] Butler M.F., Donald A.M., Ryan A.J. (1998). Time resolved simultaneous small- and wide-angle X-ray scattering during polyethylene deformation—II. Cold drawing of linear polyethylene. Polymer.

[44] Pawlak A., Galeski A. (2005). Plastic Deformation of Crystalline Polymers: The Role of Cavitation and Crystal Plasticity. Macromolecules.

[45] Pawlak A., Galeski A. (2010). Cavitation and morphological changes in polypropylene deformed at elevated temperatures. J. Polym. Sci. Part B Polym. Phys..

[46] Maksimkin A.V., Kharitonov A.P., Nematulloev S.G., Kaloshkin S.D., Gorshenkov M.V., Chukov D.I., Shchetinin I.V. (2017). Fabrication of oriented UHMWPE films using low solvent concentration. Mater. Des..

[47] Shen L., Severn J., Bastiaansen C.W.M. (2018). Drawing behavior and mechanical properties of ultra-high molecular weight polyethylene blends with a linear polyethylene wax. Polymer.

[48] Peterlin A. (1971). A Molecular model of drawing polyethylene and polypropylene. J. Mater. Sci..

[49] Wunderlich B., Czornyj G. (1977). A study of equilibrium melting of polyethylene. Macromolecules.

[50] Reinitz S.D., Carlson E.M., Levine R.A.C., Franklin K.J., Citters D.W.V. (2015). Dynamical mechanical analysis as an assay of cross-link density of orthopaedic ultra-high molecular weight polyethylene. Polym. Test..

[51] Maksimkin A.V., Nematulloev S.G., Chukov D.I., Danilov V.D., Senatov F.S. (2017). Bulk Oriented UHMWPE/FMWCNT Films for Tribological Applications. Polymers.

[52] GUR Trademark for a Group of Ultrahigh-Molecular-Weight Polyethylene Products (PE-UHMW) Produced by Ticona. https://u-hubs.com/wp-content/uploads/2019/10/GUR-PE-UHMW-Material-characteristics.pdf.

[53] Smith P., Lemstra P.J., Booij H.C. (1981). Ultradrawing of high-molecular-weight polyethylene cast from solution. II. Influence of initial polymer concentration. J. Polym. Sci. Polym. Phys. Ed..

[54] Eckstein A., Suhm J., Friedrich C., Maier R.-D., Sassmannshausen J., Bochmann M., Mülhaupt R. (1998). Determination of Plateau Moduli and Entanglement Molecular Weights of Isotactic, Syndiotactic, and Atactic Polypropylenes Synthesized with Metallocene Catalysts. Macromolecules.

[55] Strobl G. (2009). Colloquium: Laws controlling crystallization and melting in bulk polymers. Rev. Mod. Phys..

[56] Waghmare A.K., Sahoo P. (2015). Adhesive friction at the contact between rough surfaces using n-point asperity model. Eng. Sci. Technol. Int. J..

[57] Deleanu L., Botan M., Georgescu C. (2020). Tribological Behavior of Polymers and Polymer Composites.

[58] Czikos H., Czikos H. (1978). Tribology—A System Approach to the Science and Technology of Friction, Lubrication and Wear.

[59] Smirnov A., Peretyagin P., Pinargote N.W.S., Gershman I., Bartolomé J.F. (2019). Wear Behavior of Graphene-Reinforced Alumina–Silicon Carbide Whisker Nanocomposite. Nanomaterials.

[60] Liu J., Zhu C., Li G. (2020). Effect of Graphene/Graphene Oxide on Wear Resistance and Thermal Conductivity of Co-Ni Coatings. JOM.

[61] Briscoe B. (1981). Wear of polymers: An essay on fundamental aspects. Tribol. Int..

