# Supplementary material for: Structural, Mechanical, and Tribological Properties of Oriented Ultra-High Molecular Weight Polyethylene/Graphene Nanoplates/Polyaniline Films
Source: Polymers (Basel). 2023 Feb 2;15(3):758. doi: 10.3390/polym15030758 (PMC9919961; doi:10.3390/polym15030758)
Supplement: Supplementary file 1 [file polymers-15-00758-s001.zip › polymers-2160238-supplementary.pdf]

polymers

MDPI

Supplementary

Structural, Mechanical, and Tribological Properties of Oriented Ultra-High Molecular Weight Polyethylene/Graphene Nanoplates/Polyaniline Films

Tarek Dayyoub [1,2,*], Aleksey Maksimkin [2,*], Leonid K. Olifirov [1], Dilus Chukov [1], Evgeniy Kolesnikov [1], Sergey D. Kaloshkin [1] and Dmitry V. Telyshev [2,3]

[1] Department of Physical Chemistry, National University of Science and Technology "MISIS", 119049 Moscow, Russia; leonidolifirov@gmail.com (L.K.O.); dil_chukov@mail.ru (D.C.); kea.misis@gmail.com (E.K.); kaloshkin@misis.ru (S.D.K.)

[2] Institute for Bionic Technologies and Engineering, I.M. Sechenov First Moscow State Medical University (Sechenov University), Bolshaya Pirogovskaya Street 2-4, 119991 Moscow, Russia; telyshev@bms.zone

[3] Institute of Biomedical Systems, National Research University of Electronic Technology, Zelenograd, 124498 Moscow, Russia

* Correspondence: tarekzd@windowslive.com (T.D.); aleksey_maksimkin@mail.ru (A.M.)

Citation: Dayyoub, T.; Maksimkin, A.; Olifirov, L.K.; Chukov, D.; Kolesnikov, E.; Kaloshkin, S.D.; Telyshev, D.V. Structure, Mechanical and Tribological Properties of Oriented Ultra-High Molecular Weight Polyethylene/Graphene Nanoplates/Polyaniline Films. *Polymers* **2023**, *15*, x. https://doi.org/10.3390/xxxxx

Academic Editor(s): Amitesh Maiti

Received: date
Revised: date
Accepted: date
Published: date

Copyright: © 2023 by the authors. Submitted for possible open access publication under the terms and conditions of the Creative Commons Attribution (CC BY) license (https://creativecommons.org/licenses/by/4.0/).

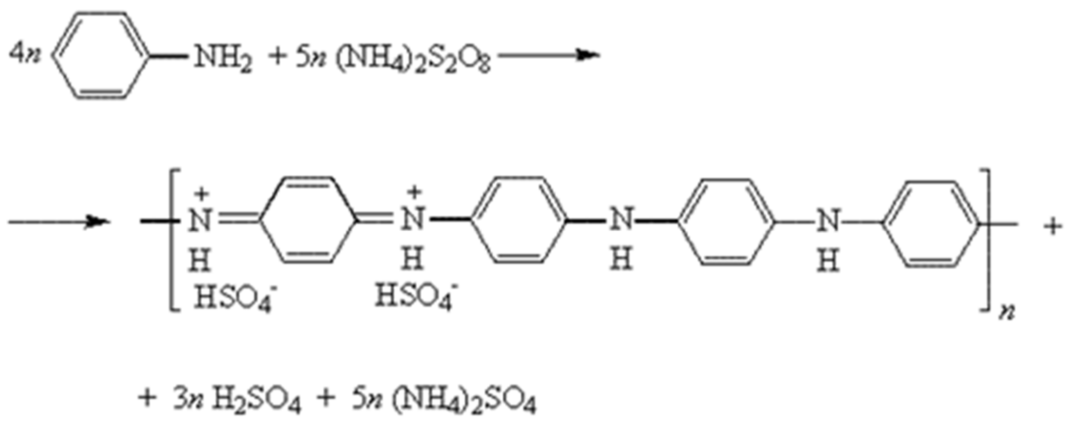

Figure S1. The scheme of oxidative polymerization of aniline [20].

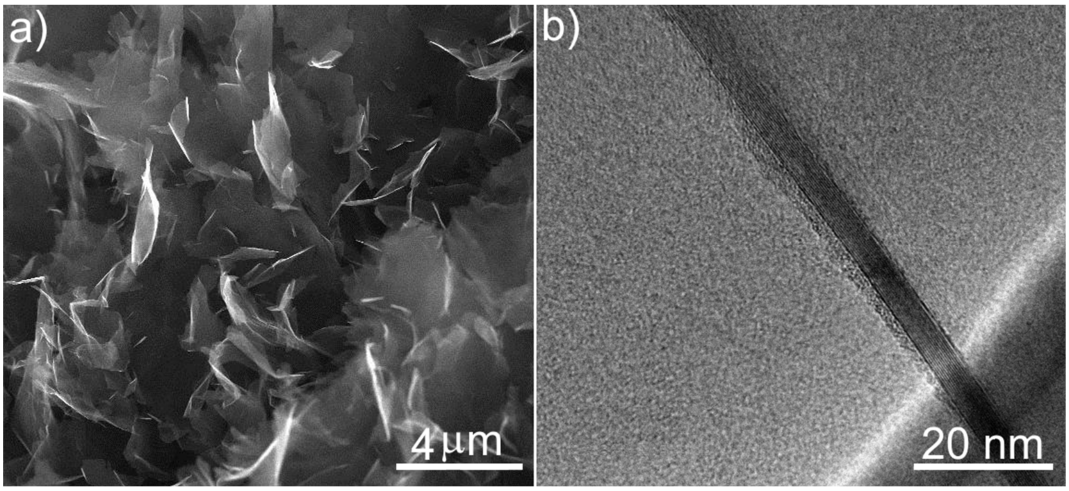

Figure S2. (**a**) SEM and (**b**) TEM micrographs of GNP microstructure [20].

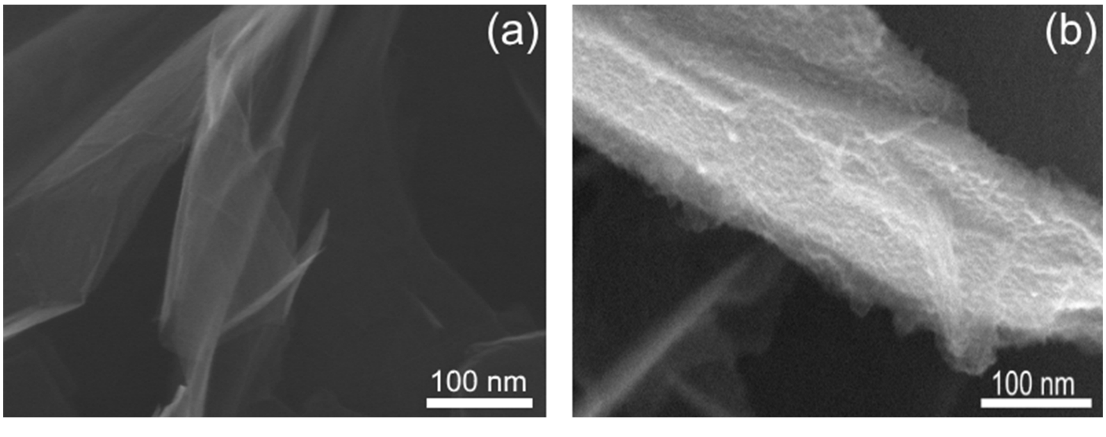

Figure S3. SEM micrograph of the (**a**) unmodified GNP and (**b**) modified GNP by PANI [20].

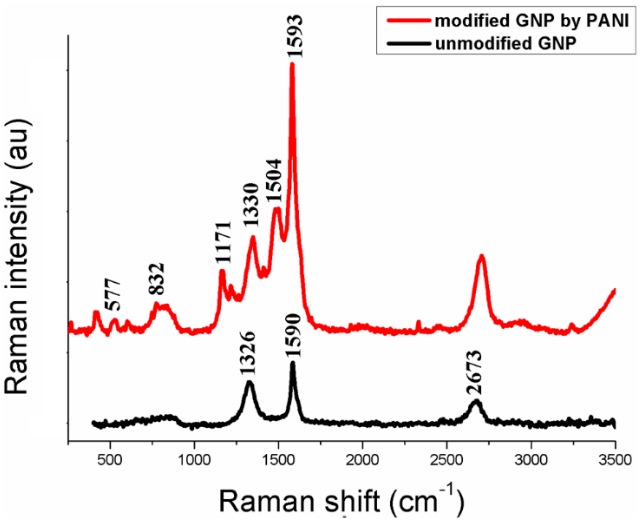

Figure S4. Raman spectra of (**a**) unmodified GNP and (**b**) modified GNP by PANI [20].

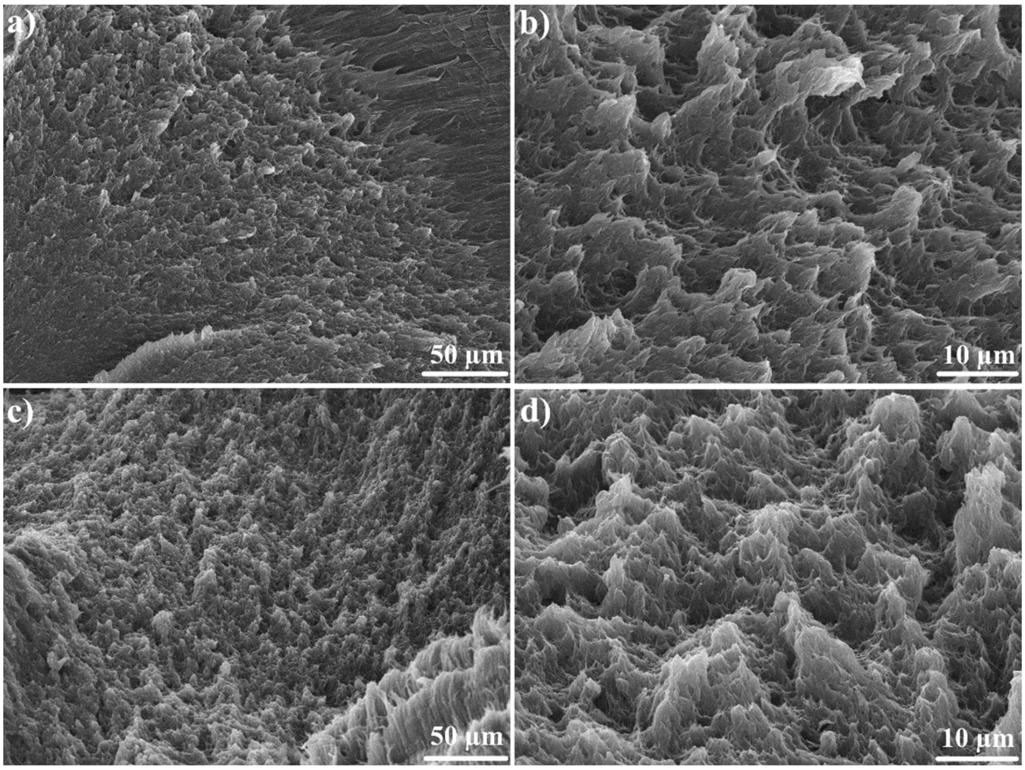

Figure S5. SEM micrographs showing typical supra-molecular structure of (**a, b**) UHMWPE /1.0 wt. % PE-wax/ 0.01 wt. % GNP/PANI, and (**c, d**) UHMWPE /1.0wt. % PE-wax/ 2.0 wt.% GNP/PANI xerogels.

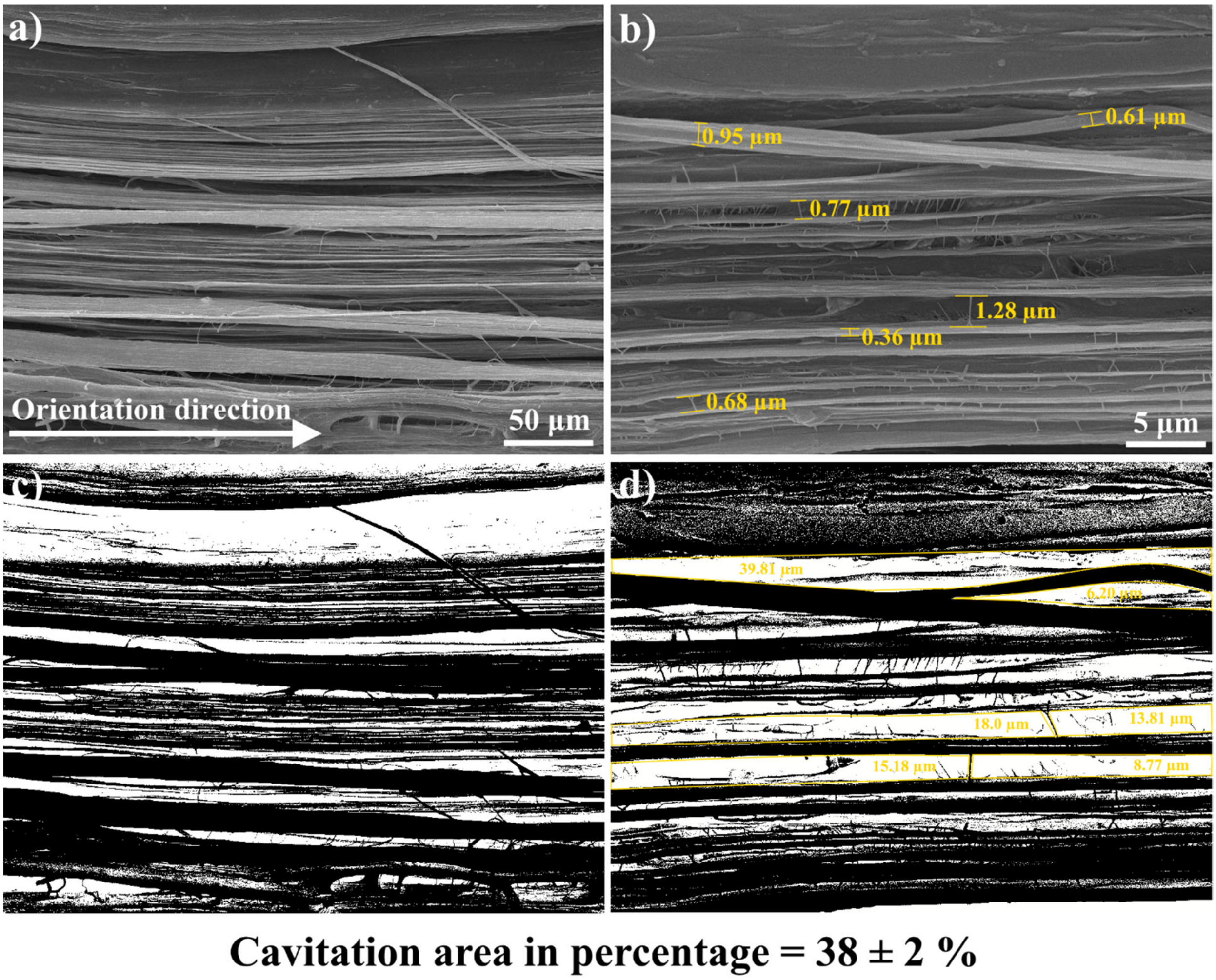

Figure S6. SEM micrographs of fibrillar structure for virgin UHMWPE films obtained by first thermal orientation regime (**a, b**), (**c, d**) modified SEM images using the image J program. The average pore size is 1.2±0.2 µm.

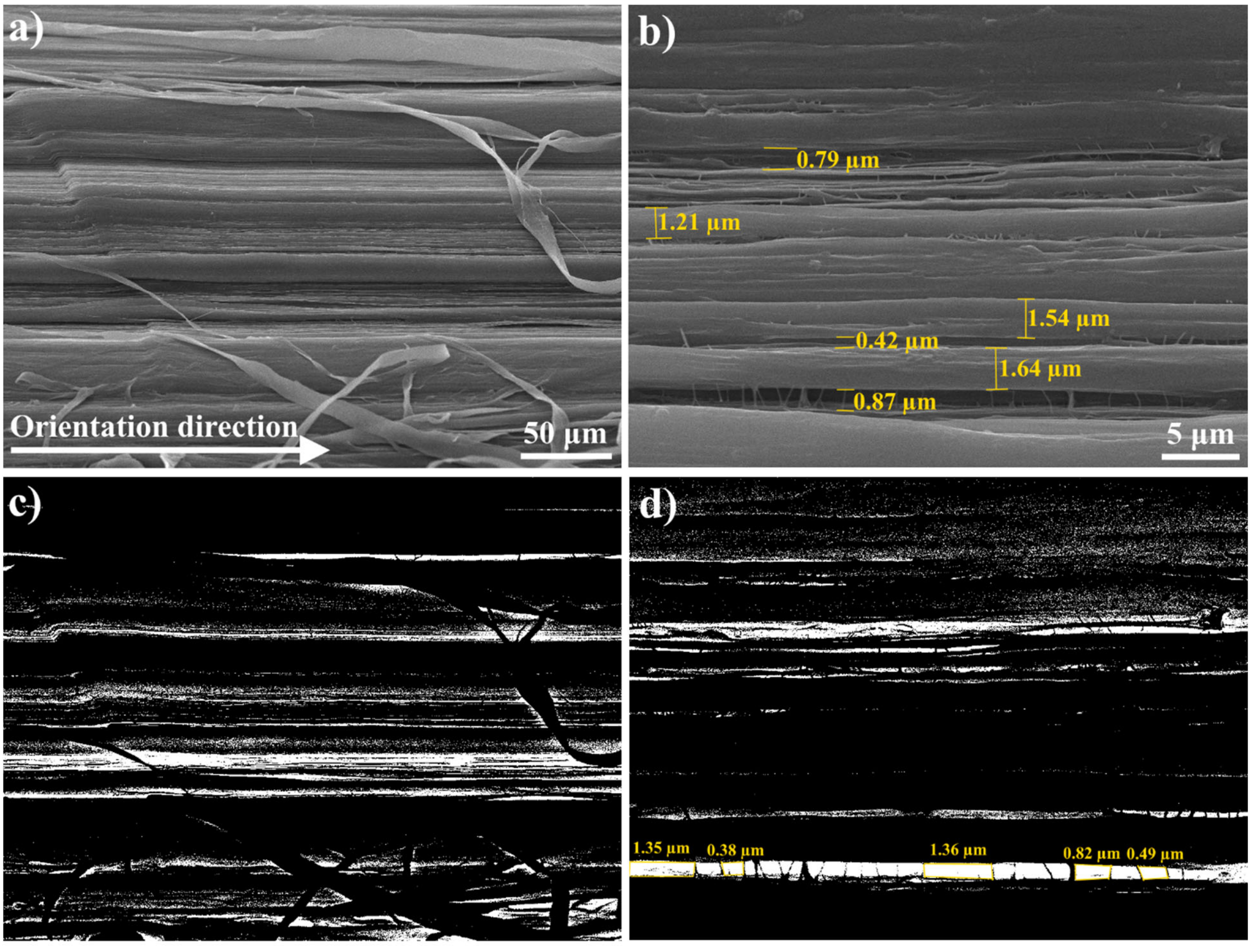

Cavitation area in percentage = 10 ± 1 %

Figure S7. SEM micrographs of fibrillar structure for UHMWPE/1.0 wt. % PE-wax films obtained by first thermal orientation regime (**a, b**), (**c, d**) modified SEM images using the image J program. The average pore size is 0.8±0.2 µm.

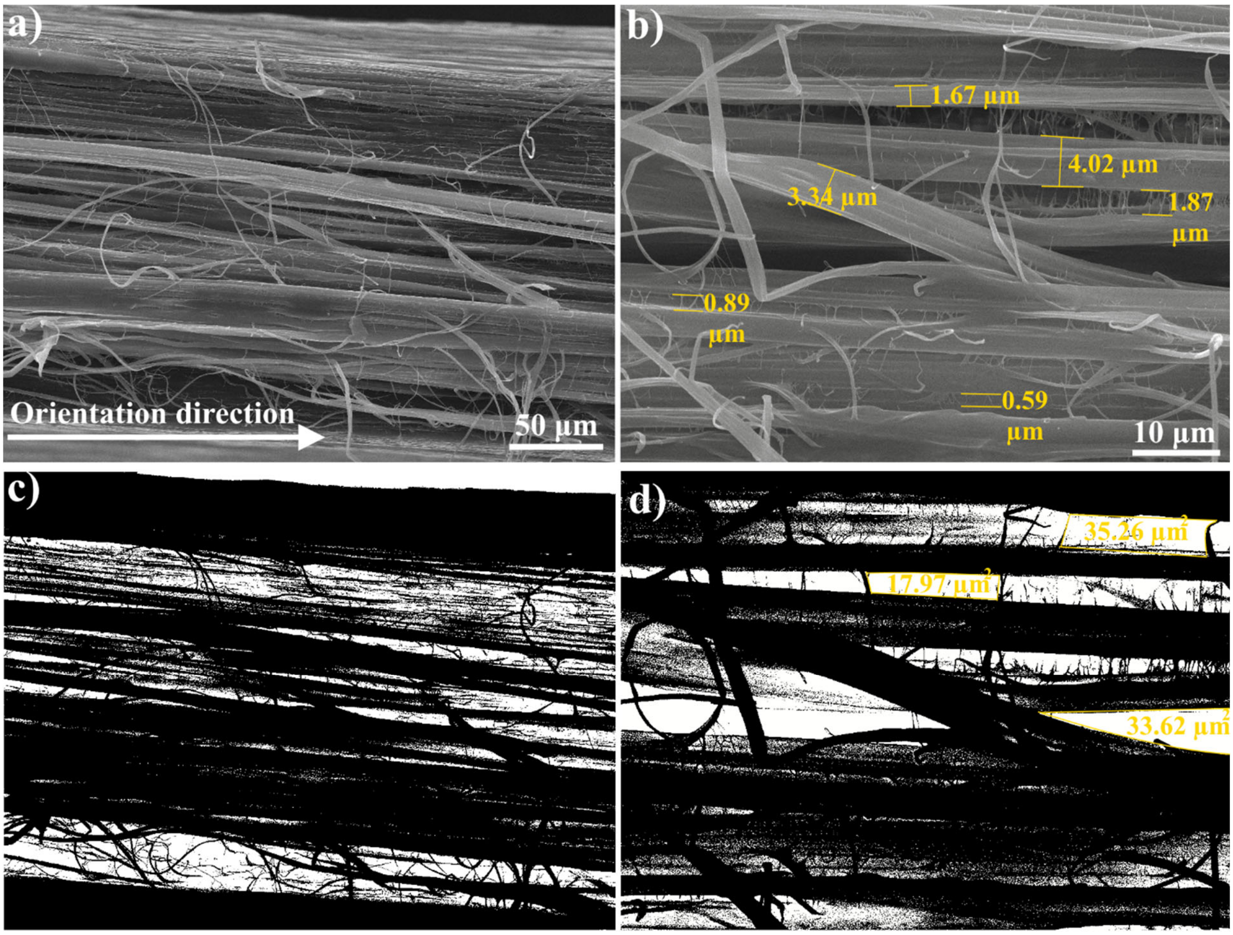

Cavitation area in percentage = 22 ± 2 %

Figure S8. SEM micrographs of fibrillar structure for UHMWPE /1.0 wt. % PE-wax/ 0.01 wt. % GNP/PAN films obtained by first thermal orientation regime (**a, b**), (**c, d**) modified SEM images using the image J program. The average pore size is 3.4±0.5 µm.

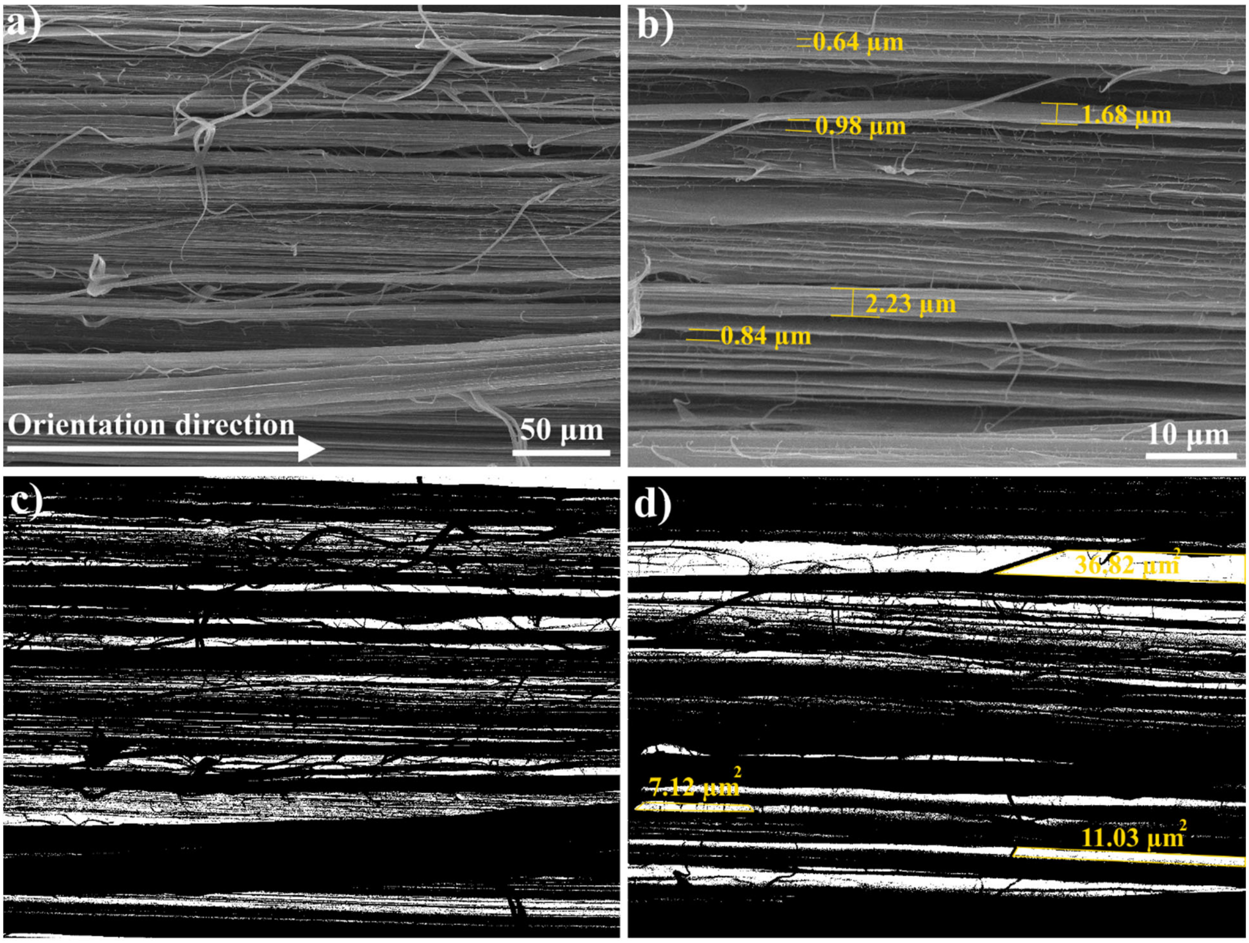

Cavitation area in percentage = 17 ± 1 %

Figure S9. SEM micrographs of fibrillar structure for UHMWPE /1.0 wt. % PE-wax/ 0.01 wt. % GNP/PAN films obtained by second thermal orientation regime (**a, b**), (**c, d**) modified SEM images using the image J program. The average pore size is 1.8±0.2 µm.

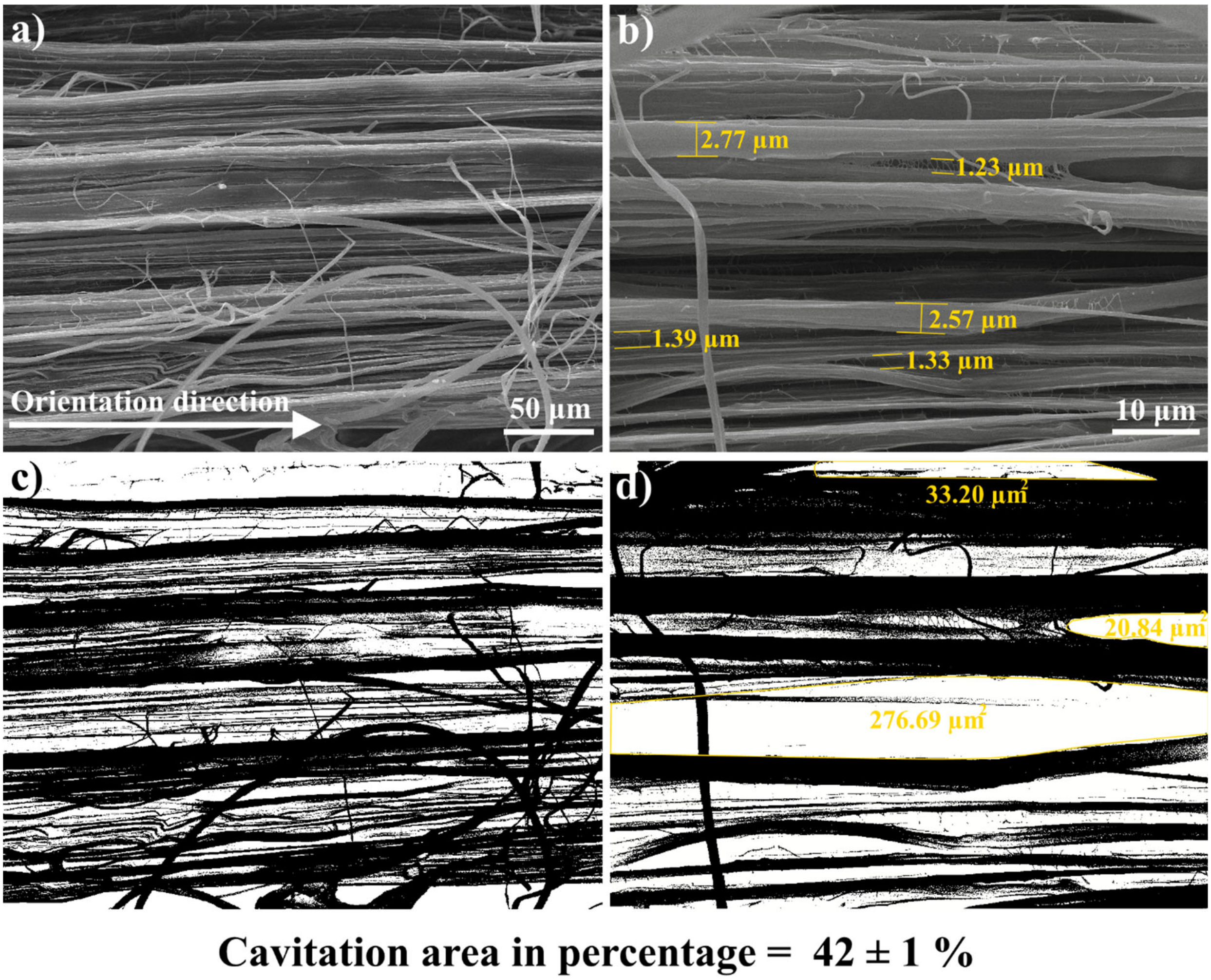

Figure S10. SEM micrographs of fibrillar structure for UHMWPE /1.0 wt. % PE-wax/ 2.0 wt. % GNP/PANI films obtained by first thermal orientation regime (**a, b**), (**c, d**) modified SEM images using the image J program. The average pore size is 3.7±0.4 µm.

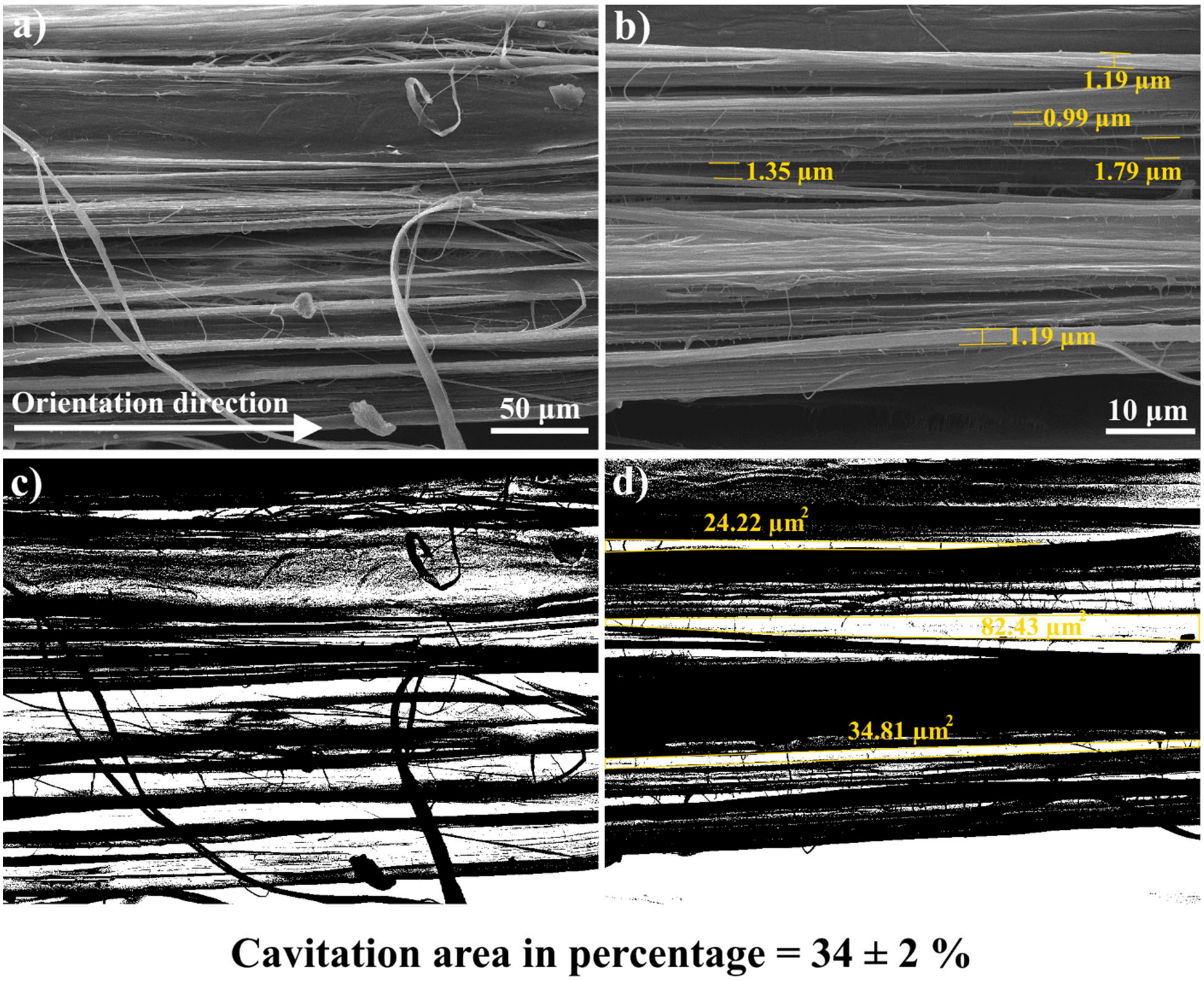

Figure S11. SEM micrographs of fibrillar structure for UHMWPE /1.0 wt. % PE-wax/ 2.0 wt. % GNP/PAN films obtained by second thermal orientation regime (**a, b**), (**c, d**) modified SEM images using the image J program. The average pore size is 2.2±0.3 µm.
